# Characterizing the cellular and molecular variabilities of peripheral immune cells in healthy recipients of BBIBP-CorV inactivated SARS-CoV-2 vaccine by single-cell RNA sequencing

**DOI:** 10.1080/22221751.2023.2187245

**Published:** 2023-05-09

**Authors:** Renyang Tong, Lingjie Luo, Yichao Zhao, Mingze Sun, Ronghong Li, Jianmei Zhong, Yifan Chen, Liuhua Hu, Zheng Li, Jianfeng Shi, Yuyan Lyu, Li Hu, Xiao Guo, Qi Liu, Tian Shuang, Chenjie Zhang, Ancai Yuan, Lingyue Sun, Zheng Zhang, Kun Qian, Lei Chen, Wei Lin, Alex F. Chen, Feng Wang, Jun Pu

**Affiliations:** a Division of Cardiology, Shanghai Immune Therapy Institute, Renji Hospital, School of Medicine, Shanghai Jiao Tong University, Shanghai, People’s Republic of China; b Shanghai Institute of Immunology, Department of Immunology and Microbiology, State Key Laboratory of Oncogenes and Related Genes, School of Medicine, Shanghai Jiao Tong University, Shanghai, People’s Republic of China; c Institute for Hepatology, National Clinical Research Center for Infectious Disease, Shenzhen Third People’s Hospital; The Second Affiliated Hospital, School of Medicine, Southern University of Science and Technology, Shenzhen, People’s Republic of China; d School of Biomedical Engineering, and Med-X Research Institute, Shanghai Jiao Tong University, Shanghai, People’s Republic of China; e Institute for Developmental and Regenerative Cardiovascular Medicine, Xinhua Hospital, Shanghai Jiao Tong University School of Medicine, Shanghai, People’s Republic of China

**Keywords:** Inactivated SARS-CoV-2 vaccine, BBIBP-CorV, single-cell RNA sequencing, single-cell TCR/BCR sequencing, peripheral blood mononuclear cells

## Abstract

Over 3 billion doses of inactivated vaccines for severe acute respiratory syndrome coronavirus 2 (SARS-CoV-2) have been administered globally. However, our understanding of the immune cell functional transcription and T cell receptor (TCR)/B cell receptor (BCR) repertoire dynamics following inactivated SARS-CoV-2 vaccination remains poorly understood. Here, we performed single-cell RNA and TCR/BCR sequencing on peripheral blood mononuclear cells at four time points after immunization with the inactivated SARS-CoV-2 vaccine BBIBP-CorV. Our analysis revealed an enrichment of monocytes, central memory CD4^+^ T cells, type 2 helper T cells and memory B cells following vaccination. Single-cell TCR-seq and RNA-seq comminating analysis identified a clonal expansion of CD4^+^ T cells (but not CD8^+^ T cells) following a booster vaccination that corresponded to a decrease in the TCR diversity of central memory CD4^+^ T cells and type 2 helper T cells. Importantly, these TCR repertoire changes and CD4^+^ T cell differentiation were correlated with the biased VJ gene usage of BCR and the antibody-producing function of B cells post-vaccination. Finally, we compared the functional transcription and repertoire dynamics in immune cells elicited by vaccination and SARS-CoV-2 infection to explore the immune responses under different stimuli. Our data provide novel molecular and cellular evidence for the CD4^+^ T cell-dependent antibody response induced by inactivated vaccine BBIBP-CorV. This information is urgently needed to develop new prevention and control strategies for SARS-CoV-2 infection. (ClinicalTrials.gov Identifier: NCT04871932).

## Introduction

As of June 2022, the World Health Organization (WHO) had reported 532 million coronavirus disease 2019 (COVID-19) infections and 6.3 million deaths worldwide (https://covid19.who.int/). The promotion and popularization of vaccination is still vital to control the spread of the disease [1]. Multiple severe acute respiratory syndrome coronavirus 2 (SARS-CoV-2) vaccines have been developed, including the inactivated vaccines BBIBP-CorV [2,3] and CoronaVac [4], mRNA vaccines BNT162b2 [5] and mRNA-1273 [6], adenovirus vaccines AZD1222 [7] and Ad5-nCoV [8], and protein subunit vaccines NVX-2373 [9] and ZF2001 [10]. To date, over 10 billion SARS-CoV-2 vaccines have been administered globally [11], over 3 billion of which were inactivated vaccines (https://ourworldindata.org/). Notably, evidence from real-world studies suggested that different vaccines, including BBIBP-CorV5, CoronaVac, mRNA and Ad5-nCoV, had distinct protection rates against severe disease [12–15]. However, the immune responses induced by different SARS-CoV-2 vaccines and their immunological mechanisms remain poorly understood. Addressing these questions requires a comprehensive understanding of host responses to these different vaccines.

Single-cell RNA sequencing (scRNA-seq) and single-cell T cell receptor (TCR)/B cell receptor (BCR) sequencing (scTCR/BCR-seq) techniques are powerful in cell biology research at single-cell resolution [16]. The recent pandemic of COVID-19 has prompted the demand for single-cell studies to uncover the immune response characteristics of different immune cells during symptom development [17]. Recent studies explored the individual immune responses to mRNA-based vaccines (BNT162b2) [18] and adenovirus vaccines (Ad5-nCoV) [19] across the transcriptome at single-cell resolution. These published scRNA-seq datasets indicated various individual immune dynamics induced by different vaccines. Along with the support of clinical practice, traditional inactivated vaccines play an important role in the prevention and control of infectious diseases [20]. Two types of inactivated SARS-CoV-2 vaccines, BBIBP-CorV and CoronaVac, have been developed, with BBIBP-CorV offering a higher protection rate against severe disease compared with CoronaVac in clinical trials [13]. A recent scRNA-seq study that focused on the CoronaVac vaccine reported potential CoronaVac-related side effects and systemic inflammation [21]. However, the functional transcription and TCR/BCR repertoire dynamics of immune cells induced by the SARS-CoV-2 vaccine, and the underlying cellular and molecular mechanism of the host adaptive immune response to the inactivated vaccine have not been well defined.

Here, by combined analysis of scRNA-seq and scTCR/BCR-seq, we revealed the unique dynamic changes in the immune response at different time points after immunization with the inactivated SARS-CoV-2 vaccine BBIBP-CorV. We also compared the transcription and repertoire dynamics of immune cells from peripheral blood mononuclear cells (PBMCs) during BBIBP-CorV vaccination and COVID-19 progression at single-cell resolution. Our study provides novel insights illustrating the possible cellular and molecular mechanisms of the post-vaccination response and facilitates an in-depth understanding of the immune responses under different stimuli.

## Materials and methods

### Participants and ethics

All human samples used in this study were processed under Institutional Review Board approved protocols at Shanghai Jiao Tong University. All study participants were recruited after providing informed consent and with the approval of the Ethics Committee of Ren Ji Hospital (KY2021-046), School of Medicine, Shanghai Jiao Tong University, and the study was conducted according to the criteria set by the Declaration of Helsinki (2013) [22]. This study was registered with ClinicalTrials.gov (NCT04871932). Written informed consent letters were routinely obtained from all participants in the study and the investigators complied with all relevant ethical regulations regarding human research participants. Nine healthy, non-frail, individuals who had not received any vaccines over the past year were recruited, including five males and four females (aged 27–66 years). All participants tested negative for SARS-CoV-2 infection by RT-qPCR tests, and tested negative for serum SARS-CoV-2-specific IgM/IgG-antibody by an ELISA before vaccination. The participants had no history of epidemiological contact with patients with COVID-19. All participants were free of clinical symptoms including fever, cough, headache, sore throat, malaise, loss of smell, runny nose, abdominal pain, diarrhea, joint pain, wheezing or dyspnea for the 28 days prior to vaccination. Participants were also free of any steroid usage, chronic diseases, pregnancy, lactation and obvious allergies to any known ingredients contained in the inactivated vaccine.

### Vaccine and vaccination protocol

For the inactivated SARS-CoV-2 vaccine (Vero cells), a single-dose schedule of 4 μg/0.5 ml was supported by the Beijing Institute of Biological Products (Beijing, China). Vaccine recipients received two doses of BBIBP-CorV via the intramuscular route, with 2–4 weeks between each injection, in accordance with the manufacturer’s instructions.

### Sample collection, preparation and storage

Peripheral venous blood samples were collected in Vacutainer CPT (362761, BD, San Diego, CA, US) tubes and coagulation promoting tubes at four time points, including the day before vaccination (BV), 7 days after the first dose (1V7), 7 days after the second dose (2V7) and 14 days after the second dose (2V14). The blood samples were processed in 2 h after collection. The PBMCs were isolated using Vacutainer CPT tubes according to the manufacturer’s protocol [23]. The viability of PBMCs in each sample was confirmed to be >90% by Trypan Blue staining. PBMCs were immediately used for scRNA library construction and sequencing, or were cryopreserved at −80°C in 10% dimethylsulfoxide in fetal bovine serum. Blood samples in the coagulation promoting tubes were incubated upright for 20 min at 4°C. Then, the coagulation promoting tubes were centrifuged at 1000 × g for 10 min at 4°C. The supernatant serum in each tube was extracted and stored at −80°C for future assays.

### Antibody and serum cytokine detection

The anti-SARS-CoV-2 (2019-nCoV) Spike RBD IgG levels in serum were measured using the SARS-CoV-2 Spike RBD Antibody Titer Assay kit (KIT002, Sino Biological, Beijing, China) following the manufacturer’s instructions. The intensity of the colour was measured at 450 nm using a MultiSkan FC reader (51119000, Thermo Fisher Scientific Inc., Waltham, MA, USA). The anti-SARS-Cov-2 RBD neutralizing antibody were detected using SARS-CoV-2 Neutralizing Antibodies Test Kit (DD3101, Vazyme Biotech, Nanjing China) according to the manufacturer’s protocol as previously reported [24], which is based on the competition between the neutralizing antibodies in serum and ACE2 to horseradish peroxidase-labelled RBD protein. The optical density (OD) was measured at 450 nm using a MultiSkan FC reader. The signal inhibition rate (SIR) was calculated with the equation SIR = (1-OD of sample/mean OD of negative control) × 100% [24]. The serum levels of IFN-γ, IFN-α, interleukin (IL)−1β, IL-2, IL-4, IL-5, IL-6, IL-10, IL-17A and tumour necrosis factor (TNF)-α were measured using an Aimplex kit (P010041, CEGER, Hangzhou, China) and flow cytometry (BD FortessaX20), following the manufacturer’s instructions. The lower limit of detection of IL-1β, IL-2, IL-4, IL-5, IL-6, IL-8, IL-10, IL-12P70, tumour necrosis factor-α, Interferon (IFN)-γ and IFN-α is 2.5 pg/ml. The lower limit of detection of IL-17A is 10 pg/ml.

### scRNA-seq library construction and sequencing

Using a Single Cell 5′ Library and Gel Bead kit (10× Genomics, 1000006) and Chromium Single Cell A Chip kit (10× Genomics, 120236), the cell suspensions (600–1000 living cells per microliter, as determined by Count Star) were loaded onto a Chromium Single Cell Controller (10× Genomics) to generate single-cell gel beads in emulsion, following the manufacturer’s protocol. Approximately 12,000 cells were added to each channel and approximately 7,000 target cells were recovered. Single-cell RNA libraries were prepared using the Chromium Single Cell 5′ Reagent (10× Genomics, PN-1000263), according to the manufacturer’s instructions. Each sequencing library was generated with a unique sample index. The libraries were sequenced using the Illumina platform with a sequencing depth of at least 100,000 reads per cell and 150 bp (PE150) paired-end reads (performed by GENE SHINE, Shanghai, China).

### scRNA-seq data processing

The matrices of unique molecular identifier (UMI) counts were generated for each sample by the Cell Ranger (10× Genomics, Version 4.0.0) Pipeline coupled with the human reference version GRCh38 (10× Genomics, Version 3.0.0.) using STAR (version 2.5.1b). The raw matrix data of 17 PBMC samples from COVID-19 patients (aged 15–62 years) were downloaded from the Gene Expression Omnibus (GEO) Data Center (accession GSE15805521) [17]. The blood of these patients with confirmed COVID-19 was sampled before September 2020. These patients were infected with wild-type SARS-CoV-2 according to World Health Organization reports. Then, the expression matrix was analyzed by R software (v.3.6.0) with the DoubletFinder package (version 2.0.2), Harmony package (v.0.1.0) and Seurat package (v.3.0.0) for filtering, data normalization, dimensionality reduction and clustering. The scRNA-seq data were analyzed via the following steps: 1) To remove low-quality cells, cells with fewer than 400 genes and a mitochondrial gene ratio of greater than 10% were excluded. Genes with at least one count in more than three cells were used for the following analysis. 2) To remove potential doublets, cells with UMI counts above the mean ± 2SD were filtered out. Additionally, we applied the DoubletFinder package, with the default parameters, to identify potential doublets. 3) For each cell, we normalized the gene expression profiles using the “NormalizeData” function with the default parameters. 4) Variable genes for each sample were selected using the “FindVariableFeatures” function with the default parameters. All ribosomal, mitochondrial and immunoglobulin genes were then removed from the list. 5) Dimension reduction was performed using the “RunPCA” function to obtain the first 50 principal components, followed by integration using Harmony to correct the batch effects. 6) A shared nearest neighbour graph was constructed based on the Euclidean distance in the low-dimensional subspace spanning the selected significant principal components. Cells were clustered using the “FindClusters” function at an appropriate resolution. 7) Cells were visualized using the UMAP algorithm with the “RunUMAP” function. Using the above pipeline, we processed the scRNA-seq data of 263,624 high-quality cells from 44 samples.

### Cell-type annotation and cluster marker identification

The “FindAllMarkers” function was used to find markers for each cluster. Clusters were then preliminarily classified and annotated based on the signature gene expression of particular cell types. In brief, the first round of clustering (resolution = 0.6) identified 12 major cell types including CD4^+^ T cells (*CD3 *^+ ^*CD4*^+^), CD8+ T cells (*CD3 *^+ ^*CD8*^+^), natural killer (NK) cells with low expression of *NCAM1* (encoding CD56) (CD56l^ow^ NK, *NKG7 *^+ ^*KLRF1 *^+ ^*NCAM1*^low^), natural killer (NK) cells highly expressing *NCAM1* (CD56^high^ NK, *NKG7 *^+ ^*KLRF1 *^+ ^*NCAM1*^high^), B cells (*CD79A *^+ ^*MS4A1*^+^), plasma cells (*CD79A *^+ ^*JCHAIN *^+ ^*MZB1*^+^), CD14^+^ monocytes (*CD14 *^+ ^*S100A12*^+^), CD16^+^ monocytes (*FCGR3A *^+ ^*MS4A7*^+^), monocyte-derived dendritic cells (mDCs, *FCER1A *^+ ^*CD1C*^+^), plasmacytoid dendritic cells (pDCs, *LILRA4*^+^), megakaryocytes (*PPBP *^+ ^*PF4*^+^) and proliferative cells (*MKI67*^+^). To identify clusters within T and B cells, we performed a second round of clustering on T cells and B/plasma cells separately. The procedure for the second round of clustering was the same as for the first round, starting from the low-rank harmony output on the highly variable genes selected as described above, with resolution ranging from 0.3–1.2. Single cells expressing two sets of well-studied canonical markers of major cell types were labelled as doublets and excluded from the following analysis. The “FindAllMarkers” function was used to find markers for each cluster. Annotation of the resulting clusters to cell types was based on the known markers. For T cells, 15 subsets were defined including seven subsets of CD4^+^ T cells, namely, naïve CD4^+^ T cells (CD4_Naive_CCR7, *CD3E ^+ ^CD4 ^+ ^CCR7 ^+ ^LEF1 ^+ ^SELL^+^*), type 1 helper T (Th1)-like cells (CD4_Th_STAT1, *CD3E ^+ ^CD4 ^+ ^ISG15 ^+ ^STAT1^+^*), type 2 helper T (Th2)-like cells (CD4_Th_GATA3, *CD3E ^+ ^CD4 ^+ ^CD40LG ^+ ^GATA3^+^*), regulatory CD4^+^ T cells (CD4_Treg_FOXP3, *CD3E ^+ ^CD4 ^+ ^FOXP3^+^*), central memory CD4^+^ T cells (CD4_Tcm_AQP3, *CD3E ^+ ^CD4 ^+ ^CCR7 ^+ ^SELL ^+ ^AQP3 ^+ ^GPR183^+^*), effector memory CD4^+^ T cells (CD4_Tem_ANXA1, *CD3E ^+ ^CD4 ^+ ^CCR7^-^SELL^-^CCR6 ^+ ^ANXA1 ^+ ^GPR183^+^*), effector CD4^+^ T cells expressing cytotoxicity gene *GNLY* (CD4_Effector_GNLY, *CD3E ^+ ^CD4 ^+ ^CCR7^-^SELL^-^GNLY^+^*); seven subsets of CD8^+^ T cells, namely, naïve CD8^+^ T cells (CD8_Naive_LEF1, *CD3E ^+ ^CD8A ^+ ^CCR7 ^+ ^LEF1 ^+ ^SELL^+^*), effector CD8^+^ T cells highly expressing *GZMH* (*CD3E ^+ ^CD8A ^+ ^CCR7^-^SELL^-^GZMH ^+ ^TYROBP^-^*), effector CD8^+^ T cells highly expressing *GNLY* (*CD3E ^+ ^CD8A ^+ ^CCR7^-^SELL^-^GNLY ^+ ^TYROBP^+^*), effector CD8^+^ T cells highly expressing *GZMK* (*CD3E ^+ ^CD8A^+^ CCR7^-^SELL^-^GZMK^+^*), mucosal-associated invariant CD8^+^ T cells (CD8_MAIT_SLC4A10, *CD3E ^+ ^CD8A ^+ ^TRAV1-2 ^+ ^SLC4A10^+^*), tissue resident memory CD8^+^ T cells (CD8_Trm_ZNF683, *CD3E ^+ ^CD8A ^+ ^ZNF683 ^+ ^KLRC2^+^*) and exhausted CD8^+^ T cells (CD8_Exhausted_HAVCR2, *CD3E ^+ ^CD8B ^+ ^HAVCR2 ^+ ^CTLA4 ^+ ^TIGIT^+^*); and one subset of γδ T cells (*CD3E ^+ ^TRDV2 ^+ ^TRGV9^+^*). For B/plasma cells, five B cell subsets were defined including naïve B cells (Naïve_B, *CD79A ^+ ^MS4A1 ^+ ^IGHD^+^*), germinal centre B cells (Germinal_central_B, *CD79A ^+ ^MS4A1 ^+ ^NEIL1^+^*), memory B cells (Memory_B, *MS4A1 ^+ ^CD27^+^*), atypical memory B cells (Atypical_memory_B, *MS4A1 ^+ ^CD27 ^+ ^CIB1^+^*) and plasma B cells (Plasma_B_XBP1, *CD79A ^+ ^CD38 ^+ ^MZB1^+^*). The list of signature genes for each cluster is provided in Supplementary Table 2.

### Identification of differential expression genes and functional enrichment analysis

Differential gene expression testing was performed using the “FindMarkers” function in Seurat with the default parameter “test.use = wilcox” and the Benjamini–Hochberg method was used to estimate the adjusted *p* value. Differential expression genes (DEGs) were filtered using a minimum log_2_ (fold-change) of 0.4 and a maximum adjusted *p* value of 0.05. Gene ontology biological process (GO:BP) functional enrichment analysis of the upregulated DEGs in the 2V7 and 2V14 groups was conducted using the R package clusterProfiler, with the default parameters. Gene Set Enrichment Analysis (GSEA) between the 2V7 and 2V14 groups and the BV group was also conducted using the R package clusterProfiler, with the default parameters.

### TCR and BCR V(D)J sequencing and analysis

TCR and BCR clonal types were determined using the CellRanger V(D)J pipeline (10× Genomics, Version 4.0.0). The TCR and BCR repertoire sequencing data were filtered according to the following criteria: 1) productive is “True”; 2) high_confidence is “True”; 3) umis ≥1; and 4) raw_clonotype_id is not “None”. To identify the TCR clonotype for each T cell, only cells with at least one TCR α chain and one TCR β chain were used. For a given T cell, if there were two or more TCR α or TCR β chains assembled, the highest expressed (UMI or reads) TCR α or TCR β chains were regarded as the dominant TCR α or TCR β chain in the cell. Each unique TCR α(s)-TCR β(s) pair (CDR3 nucleotide sequences and rearranged VJ genes included) was defined as a clonotype. BCR clonotypes were identified in a similar manner to TCR clonotypes. Only cells with at least one productive heavy chain (IGH) and one productive light chain (IGL or IGK) were kept for further analysis. Each unique IGH-IGL/IGK pair was defined as a clonotype. For a given B cell, if there were two or more IGH or IGL/IGK assembled, the highest expressed (UMI or reads) IGH or IGL/IGK chain was defined as the dominant IGH or IGL/IGK chain in the cell. If one TCR α-TCR β pair or one IGH-IGL/IGK pair was present in at least two cells, cells harbouring this clonotype were considered to be clonal expanded and the number of cells with the same clonotype indicated the degree of cellular clonal expansion.

Cells expressing the same clonotype were counted. The counts were classified into eight classes to identify the cellular clonal expansion status, i.e. > 100, 51–100, 31–50, 21–30, 11–20, 6–10, 2–5, and a unique clone without expansion. For CD4^+^ T cells, CD8^+^ T cells and B/plasma cells, the clonal expansion status percentage was computed for each group. The percentage of the TCR type count to the total cell number was used to measure TCR diversity.

### Transcription factor analysis

After arranging the input by the gene expression raw matrix, the SCENIC package in R facilitated evaluation of transcription factors among immune cell subsets using the default parameters. The pheatmap and ggplot2 packages in R were adopted to visualize the expression profile of transcription factors.

### Definition of cell state scores

For assessing the immune state, the “AddModuleScore” function in Seurat was used to calculate the modular score of pathways with default settings. The following gene sets, positive regulation of type I interferon production (GO:0032481), T cell receptor signalling pathway (GO:0050852), immunoglobulin production (GO:0002377), interleukin-6 production (GO:0032635), tumour necrosis factor production (GO:0032640) and positive regulation of T cell cytokine production (GO:0002726) were used to define type I interferon production, T cell receptor signalling, immunoglobulin production, interleukin-6 production, tumour necrosis factor production and T cell cytokine production, respectively. The expression score for each pathway was calculated based on the average expression of genes from the abovementioned gene sets.

### Statistical analysis

Statistical analyses were performed using the R software package (version 3.6.0) and involved the Shapiro–Wilk test, F-test, Levene’s test, unpaired Mann–Whitney U-test and Kruskal–Wallis test as previously reported [25]. Data normality was determined by the Shapiro–Wilk test. The F-test was used to examine the homogeneity of variance for two group comparisons. The Levene’s test was used to examine homogeneity of variance for the Kruskal–Wallis rank sum test. We performed unpaired Mann–Whitney U-tests on the levels of cytokines in serum, cell subset proportions, the fraction of cells expressing cytokine genes and immunoglobulin heavy chain genes, the module score of gene sets, TCR diversity and the usage of BCR and TCR genes between pre- and post-vaccination samples. When comparing the usage of paired TCR and BCR genes among the four time points, the Kruskal–Wallis rank sum test was used, followed by Benjamini and Hochberg adjustment. The *p* value significance thresholds used for each set of data are described in the corresponding methods and figure legends.

### Data and code availability

The processed transcriptomic data reported in this study has been deposited in China National Center for Bioinformation, National Genomics Data Center under the accession code HRA004001, OMIX003038 and PRJCA004747. During our analysis, which proceeded as described in the methods section, we did not use any special code.

## Results

### Overview of the immunological changes after BBIBP-CorV vaccination

**Figure 1.**
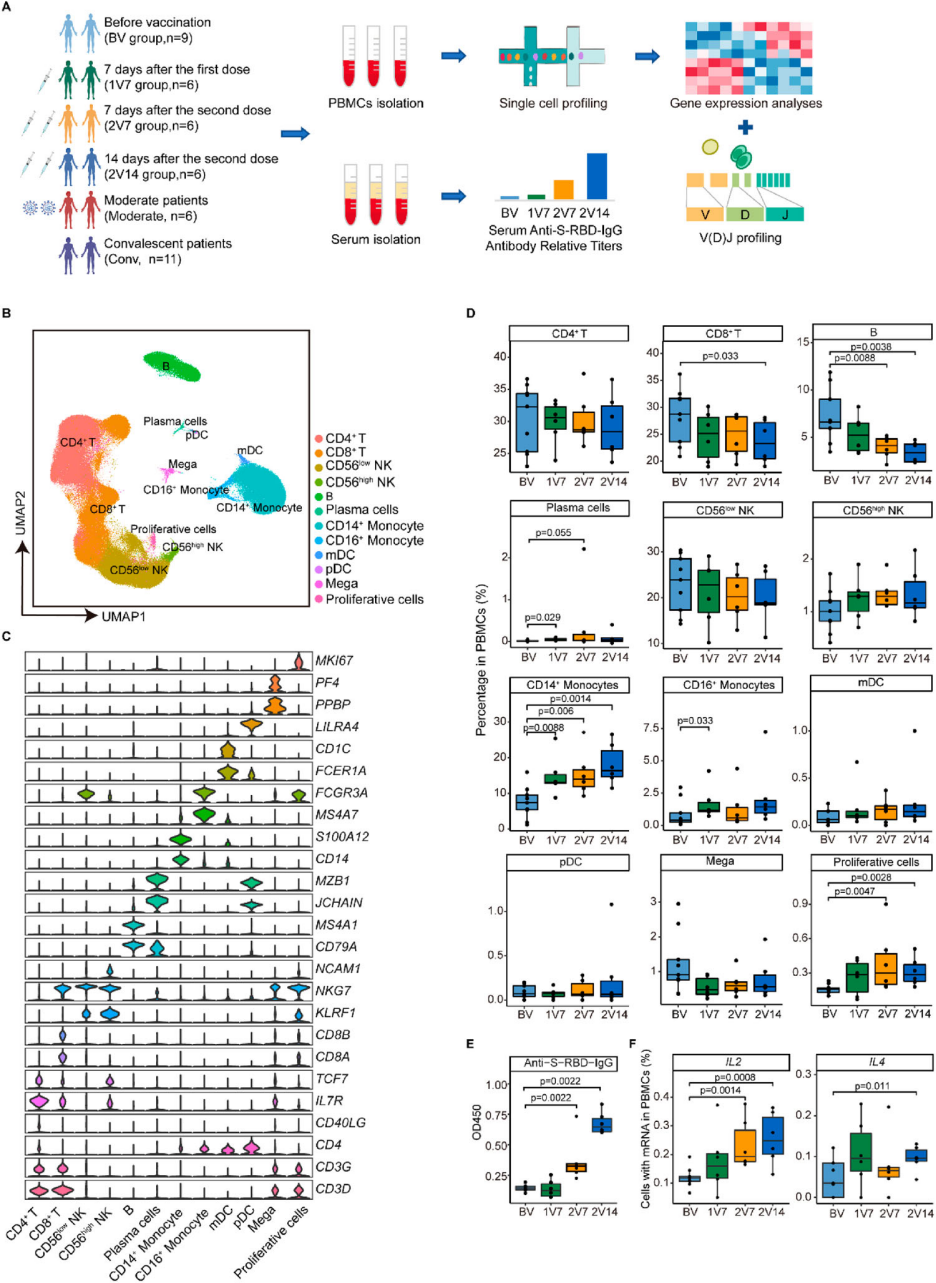
Overview of immunological changes induced by BBIBP-CorV vaccination. **A**. Workflow of vaccination, sampling and scRNA-seq. **B**. UMAP charts of 263,624 single cells coloured according to cell type. **C**. Violin plot showing the expression of signature genes in the 12 cell types. **D**. Box charts showing the proportion of each cell type in total PBMCs of each sample across four groups (n = 9 in BV group, n = 6 in the 1V7, 2V7 and 2V14 groups). All box plots display the median, 25th and 75th percentiles, and whiskers extending to the maximum and minimum data points. Data were analyzed by an unpaired Mann-Whitney U-test. A *p* value < 0.05 was considered statistically significant. **E**. Measurement of anti-recombinant S-RBD neutralizing antibodies across the four groups (n = 6 in the BV, 1V7, 2V7 and 2V14 groups). Serum was diluted 55 times for IgG. S-RBD, receptor binding domain of spike protein. **F**. Fraction of cells expressing *IL2* and *IL4* in BV group (n = 9), 1V7 group (n = 6), 2V7 group (n = 6) and 2V14 group (n = 6). All box plots display the median, 25th and 75th percentiles, and whiskers extending to the maximum and minimum data points. Data were analyzed by an unpaired Mann–Whitney U-test. A p value< 0.05 was considered statistically significant. Conv, convalescent patients. Mega, megakaryocytes. S-RBD, Spike receptor binding domain. BV group, before vaccination. 1V7 group, 7 days after the first vaccination. 2V7 group, 7 days after the second vaccination. 2V14 group, 14 days after the second vaccination.

To comprehensively assess the immune responses following vaccination with inactivated SARS-CoV-2 vaccine (BBIBP-CorV), nine healthy donors (HDs) were enrolled in this 2019-nCoV vaccine observational study [NCT04871932] according to the inclusion and exclusion criteria (Figure 1A and Supplementary Table 1). First, peripheral blood samples from the nine HDs were collected before vaccination (BV group, n = 9). Then, six of the nine HDs received two doses of BBIBP-CorV vaccine. Peripheral blood samples from all two-dose-vaccinated participants were collected 7 days after the first vaccination (1V7 group, n = 6), 7 days after the second vaccination (2V7 group, n = 6) and 14 days after the second vaccination (2V14 group, n = 6) (Figure 1A). Serum and PBMCs were separated from all peripheral blood samples, and 2019-nCoV Spike receptor binding domain (RBD) antibodies and cytokines in serum were measured. After isolating the PBMCs, we profiled the V(D)J repertoires of T and B cells integrated with 5’ Gene Expression using a droplet-based single-cell sequencing method (10×Genomics) (Figure 1A). In addition, to reveal differences in the immune response between vaccination and SARS-CoV-2 infection, we collected 17 published PBMCs data [17] with single-cell RNA and TCR/BCR sequencing from patients with confirmed COVID-19 of both sexes. Among these 17 samples, 6 were from COVID-19-infected patients with moderate symptoms and 11 were from recovered convalescent patients (Figure 1A). Finally, 44 samples were classified according to six clinical conditions: before vaccination (BV group, n = 9), 7 days after the first dose (1V7 group, n = 6), 7 days after the second dose (2V7 group, n = 6), 14 days after the second dose (2V14 group, n = 6), symptomatic COVID-19 patients (n = 6) and convalescent patients (conv; n = 11) (Figure 1A). The detailed clinical information is listed in Supplementary Table 1.

After filtering out low-quality droplets, 263,624 cells were obtained from 44 PBMC samples. The filtered data were integrated, dimension reduced and clustered in an unsupervised method. Based on the unsupervised clustering and expression of signature genes for each cluster (Supplementary Table 2), 12 cell types were identified (Figure 1B and S1A). These cell types included CD4^+^ T cells (*CD3 *^+ ^*CD4*^+^), CD8^+^ T cells (*CD3 *^+ ^*CD8*^+^), natural killer (NK) cells with low expression of *NCAM1* (encoding CD56) (CD56^low^ NK, *NKG7 *^+ ^*KLRF1 *^+ ^*NCAM1*^low^), NK cells highly expressing *NCAM1* (CD56^high^ NK, *NKG7 *^+ ^*KLRF1 *^+ ^*NCAM1*^high^), B cells (*CD79A *^+ ^*MS4A1*^+^), plasma cells (*CD79A *^+ ^*JCHAIN *^+ ^*MZB1*^+^), CD14^+^ monocytes (*CD14 *^+ ^*S100A12*^+^), CD16^+^ monocytes (*FCGR3A *^+ ^*MS4A7*^+^), monocyte-derived dendritic cells (mDCs, *FCER1A *^+ ^*CD1C*^+^), plasmacytoid dendritic cells (pDCs, *LILRA4*^+^), megakaryocytes (*PPBP *^+ ^*PF4*^+^) and proliferative cells (*MKI67*^+^) (Figure 1C and S1B).

To reveal the dynamics of the cell composition at different time points before and after vaccination, we calculated the relative percentage of all cell types in the PBMCs of each sample (Figure 1D). We found that CD14^+^ monocytes were significantly increased in the 1V7, 2V7 and 2V14 groups compared with the BV group (Figure 1D), which was consistent with the BBIBP-CorV vaccination study using flow cytometry [26]. The relative percentage of CD16^+^ monocytes was significantly increased in the 1V7 group compared with the BV group (Figure 1D). Both CD14^+^ monocytes and CD16^+^ monocytes have great potential for cytokine production [27], which plays a role in humoral immunity against extracellular pathogen invasion [28]. The proportion of proliferative cells was significantly increased in the 2V7 and 2V14 groups compared with the BV group (Figure 1D). The relative abundance of plasma cells was increased in the 1V7 group (Figure 1D). By contrast, the relative percentage of B cells (excluding plasma cells) decreased after the second dose (Figure 1D). CD8^+^ T cells were decreased in the 2V14 group compared with the BV group.

To further validate the vaccination efficacy in our participants, 2019-nCoV Spike RBD IgG levels and neutralizing antibodies were measured using indirect enzyme-linked immunosorbent assay (ELISA) kits. Anti-S-RBD IgG levels and neutralizing antibodies were significantly increased after the second dose vaccination (Figure 1E and S1C). Next, we calculated the fraction of cells expressing cytokine genes including *IL1B*, *IL2*, *IL4*, *IL5*, *IL6*, *IL10*, *TNF* and *IFNG*, at single-cell resolution. Compared with the BV group, the fraction of cells expressing *IL2* (a T cell growth factor involved in the clonal expansion of antigen-specific T cells [29]) in PBMCs was significantly increased in the 2V7 and 2V14 groups (Figure 1F). The fraction of cells expressing *IL4* (a crucial immune factor for the differentiation of naïve T cells into type 2 helper T cells [30]) in PBMCs was significantly increased in the 2V14 group (Figure 1F). By contrast, the fraction of cells expressing cytokine gene *IL6* in PBMCs was significantly decreased after vaccination (Figure S1D). Whereas, the serum levels of circulating pro-inflammatory cytokines, such as IL2, IL-6 and TNF-α, did not significantly change after vaccination (Figure S1E).

### Features of innate immune cells after BBIBP-CorV vaccination

**Figure 2.**
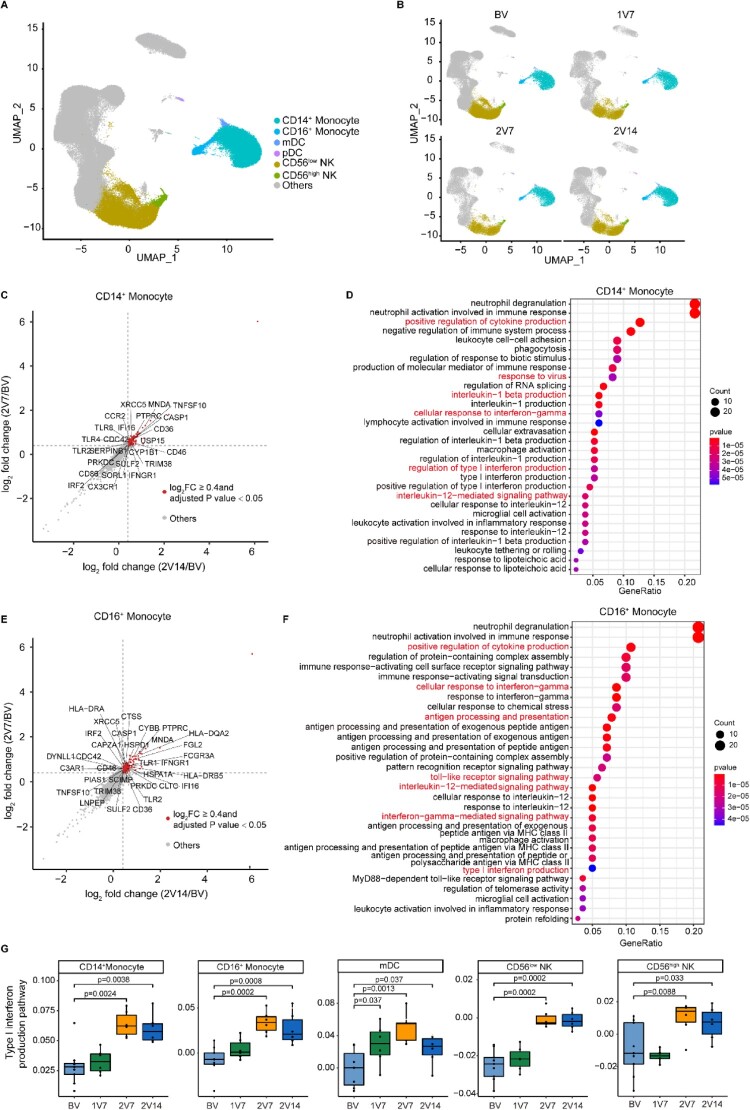
Characterization of innate immune cells after BBIBP-CorV vaccination. **A** and **B**. UMAP plots showing the innate immune cells in total (**A**) and in the four groups separately (**B**). Each dot corresponds to a single cell. Cells are coloured according to cell type. **C**. Scatter-plot showing DEGs in CD14^+^ monocytes of the 2V7 and 2V14 groups compared with those in the BV group. Each red dot denotes an individual gene with Log_2_FC ≥ 0.4 and adjusted *p* value < 0.05 for the 2V7/BV and 2V14/BV comparisons. **D**. Functional enrichment analyses of the DEGs coloured in red in the scatter-plot in **C**. The top 30 enriched BP terms are shown. Interesting BP terms are highlighted in red. **E**. Scatter-plot showing DEGs in CD16^+^ monocytes of the 2V7 and 2V14 groups compared with those in the BV group. Each red dot denotes an individual gene with Log_2_FC ≥ 0.4 and adjusted *p* value < 0.05 for the 2V7/BV and 2V14/BV comparisons. **F**. Functional enrichment analyses of the DEGs coloured in red in the scatter-plot in **E**. The top 30 enriched BP terms are shown. Interesting BP terms are highlighted in red. **G**. Box plots showing the expression of the type I interferon production pathway across innate immune cells derived from BV group (n = 9), 1V7 group (n = 6), 2V7 group (n = 6) and 2V14 group (n = 6). Groups are shown in different colours. All box plots display the median, 25th and 75th percentiles, and whiskers extending to the maximum and minimum data points. Data were analyzed by an unpaired Mann-Whitney U-test. A p value< 0.05 was considered statistically significant. DEGs, differentially expressed genes. FC, fold change. BP, biological process. BV group, before vaccination. 1V7 group, 7 days after the first vaccination. 2V7 group, 7 days after the second vaccination. 2V14 group, 14 days after the second vaccination.

To further investigate the transcriptomic changes in innate immune cells (Figure 2A, B) after BBIBP-CorV vaccination, we compared the gene expression profiles of monocytes and NK cells pre- and post-vaccination. An immune response against the virus is generally achieved 2 weeks after booster vaccination [31]. We found that anti-S-RBD IgG levels and neutralizing antibodies were significantly increased after the second dose vaccination (Figure 1E and S1C). Thus, we focused the 2V7 and 2V14 groups for further analyses. We identified DEGs in the CD14^+^ monocytes and CD16^+^ monocytes of the 2V7 and 2V14 groups compared with the BV group (Figure 2C-F). Functional enrichment analysis showed that the upregulated DEGs in CD14^+^ monocytes in the 2V7 and 2V14 groups were enriched in “interlukin-1 production”, “positive regulation of cytokine production”, “cellular response to interferon-gamma” and “type I interferon production” pathways (Figure 2C, D). Interestingly, the IL-1 family cytokines are potential mucosal vaccine adjuvants and can induce antigen-specific immune responses against pathogens such as influenza virus [32]. The upregulated DEGs in CD16^+^ monocytes in the 2V7 and 2V14 groups were enriched in “type I interferon production”, “positive regulation of cytokine production”, “response to interferon gamma”, “toll-like receptor signaling pathway” and “antigen processing and exogenous antigen” pathways (Figure 2E, F). Notably, type I interferon signalling promotes humoral immunity following vaccination, including vaccine-induced antibody, B cell and follicular helper T (Tfh) cell responses [33]. Additionally, we analyzed the expression of type I interferon production-related pathway in innate immune cells across four time points. We found that type I interferon production was uniformly and significantly upregulated in monocytes, mDCs and NK cells in the 2V7 and 2V14 groups (Figure 2G).

In addition, we identified DEGs in NK cells, including CD56^low^ and CD56^high^ NK cells, by comparing the 2V7 and 2V14 groups with the BV group (Figure S2A, B). Functional enrichment analysis showed that upregulated DEGs in the 2V7 and 2V14 groups were enriched in the “response to virus”, “lymphocyte differentiation” and “response to interferon-gamma” pathways, which had previously been reported in COVID-19 patients [34].

### Characterization of T cells after BBIBP-CorV vaccination

**Figure 3:**
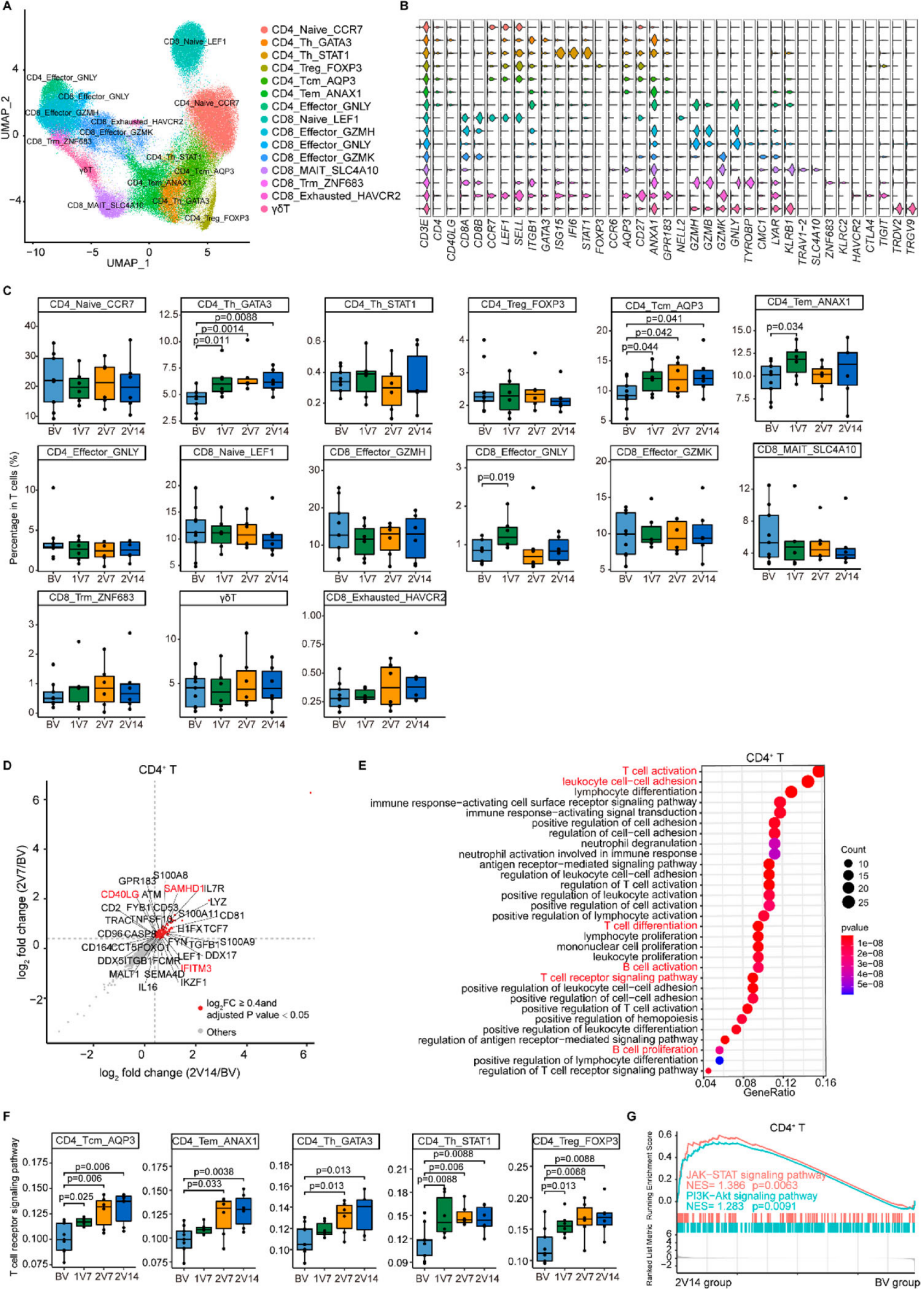
. Characterization of T cells after BBIBP-CorV vaccination. **A**. UMAP plot showing the T cells coloured according to cell subsets. **B**. Violin plots showing the expression of signature genes in 15 T cell subsets. **C.** Box plots showing the proportion of each T cell subset in total T cells of each sample across four groups (n = 9 in BV group, n = 6 in the 1V7, 2V7 and 2V14 groups). Groups are shown in different colours. All box plots display the median, 25th and 75th percentiles, and whiskers extending to the maximum and minimum data points. Data were analyzed by an unpaired Mann-Whitney U-test. A p value< 0.05 was considered statistically significant. **D**. Scatter-plot showing the DEGs in CD4^+^ T cells of the 2V7 and2V14 groups compared with those in the BV group. Each red dot denotes an individual gene with Log_2_FC ≥ 0.4 and adjusted *p* value < 0.05 for the 2V7/BV and 2V14/BV comparisons. **E**. Functional enrichment analyses of the DEGs coloured in red in the scatter-plot in **D**. The top 30 enriched BP terms are shown. Interesting BP terms are highlighted in red. **F**. Box plots showing the expression of the T cell receptor signalling pathway across CD4^+^ T cell subsets derived from BV group (n = 9), 1V7 group (n = 6), 2V7 group (n = 6) and 2V14 group (n = 6). Groups are shown in different colours. All box plots display the median, 25th and 75th percentiles, and whiskers extending to the maximum and minimum data points. Data were analyzed by an unpaired Mann-Whitney U-test. A p value< 0.05 was considered statistically significant. **G**. GSEA enrichment plots showing two upregulated gene sets in CD4^+^ T cells of the 2V14 group compared with those in the BV group. NES, normalized enrichment score. A *p* value < 0.05 was considered statistically significant. DEGs, differentially expressed genes. FC, fold change. BP, biological process. BV group, before vaccination. 1V7 group, 7 days after the first vaccination. 2V7 group, 7 days after the second vaccination. 2V14 group, 14 days after the second vaccination.

To characterize changes in T cell subsets, we subclustered T cells from PBMCs and obtained 15 subsets according to the expression of T cell signature genes (Figure 3A, B and Supplementary Table 2, see Methods). To gain insight into the features in T cell subsets before and after vaccination, we calculated the relative percentage of each T cell subset against the total T cell population of each sample (Figure 3C). The proportion of CD4_Tem_ANAX1 cells was significantly increased in the 1V7 group compared with the BV group (Figure 3C). Whereas, CD4_Th_GATA3 and CD4_Tcm_AQP3 cells expanded in the 1V7, 2V7 and 2V14 groups compared with the BV group (Figure 3C). CD4_Th_GATA3 cells harboured Th2 cell features with high expression of *GATA3* (Figure 3B), which was involved in humoral immunity against extracellular pathogens by producing cytokines such as IL-4, IL-5 and IL-13 [35,36]. The increased central memory CD4^+^ T cells (CD4_Tcm_AQP3) produce cytokines associated with rapid T cell secondary expansion [37], such as IL-2. In addition, the proportion of CD8_Effctor_GNLY cells was significantly increased in the 1V7 group compared with the BV group, and returned to basal levels after the second dose vaccination (Figure 3C).

To further investigate the transcriptomic changes in T cells after the booster vaccination, we compared the gene expression profiles of the 2V7 and 2V14 groups with that of the BV group in CD4^+^ T and CD8^+^ T cells. For CD4^+^ T cells, functional enrichment analysis showed that the upregulated DEGs in the 2V7 and 2V14 groups were enriched in “T cell activation”, “leukocyte cell–cell adhesion”, “T cell differentiation”, “T cell receptor signaling pathway” and “B cell activation” pathways (Figure 3D, E and S3A, B). In CD4^+^ T cells, the expression of IFN-related gene *IFITM3* [38] and broad-spectrum anti-retroviral factor *SAMHD1* [39] was significantly upregulated in the 2V7 and 2V14 groups compared with the BV group (Figure 3D and S3A, B). These results indicated enhanced anti-viral immunity after the booster vaccination. In addition, we analyzed the expression of T cell receptor signalling pathway in memory and Th CD4^+^ T cell subsets across four time points. We found that expression of the T cell receptor signalling pathway was significantly upregulated in these cell subsets including memory CD4^+^ T cells (CD4_Tcm_ANXA1 and CD4_Tem_ANXA1), helper CD4^+^ T cells (CD4_Th_GATA3 and CD4_Th_STAT1) and regulatory CD4^+^ T cells (CD4_Treg_FOXP3) from the 2V7 and 2V14 groups (Figure 3F). Furthermore, Gene Set Enrichment Analysis (GSEA) showed that the “positive regulation of humoral immune response” and “positive regulation of T cell cytokine production” pathways were significantly enriched in CD4^+^ T cells from the 2V7 and 2V14 groups (Figure S3C). Additionally, the JAK-STAT signalling and PI3K-Akt signalling pathways were significantly enriched in CD4^+^ T cells from the 2V14 group (Figure 3G). For CD8^+^ T cells, functional enrichment analysis showed that upregulated DEGs in CD8^+^ T cells in the 2V7 and 2V14 groups were enriched in “T cell activation”, “leukocyte cell–cell adhesion”, “T cell differentiation”, “T cell receptor signaling pathway” and “B cell activation” pathways (Figure S3D, E). Remarkably, the expression of cytotoxicity-related genes *(FCGR3A* [40] and *KLRD1* [41]) and anti-retroviral factor *SAMHD1* in CD8^+^ T cells was significantly increased in the 2V7 and 2V14 groups compared with the BV group (Figure S3D, E). The expression of granzyme-related genes (*GZMA* and *GZMK*) was upregulated in CD8^+^ T cells in the 2V7 group (Figure S3D), whereas the expression of cytotoxicity gene *PRF1* (encoding perforin 1) was significantly increased in CD8^+^ T cells in the 2V14 group compared with the BV group (Figure S3E). Collectively, these highly expressed IFN-related genes and cytotoxic genes hinted that T cells may possess powerful cytotoxicity against SARS-CoV-2-infected cells, following booster vaccination.

### Global profile of CDR3 length and TCR VJ genes distribution after BBIBP-CorV vaccination

**Figure 4.**
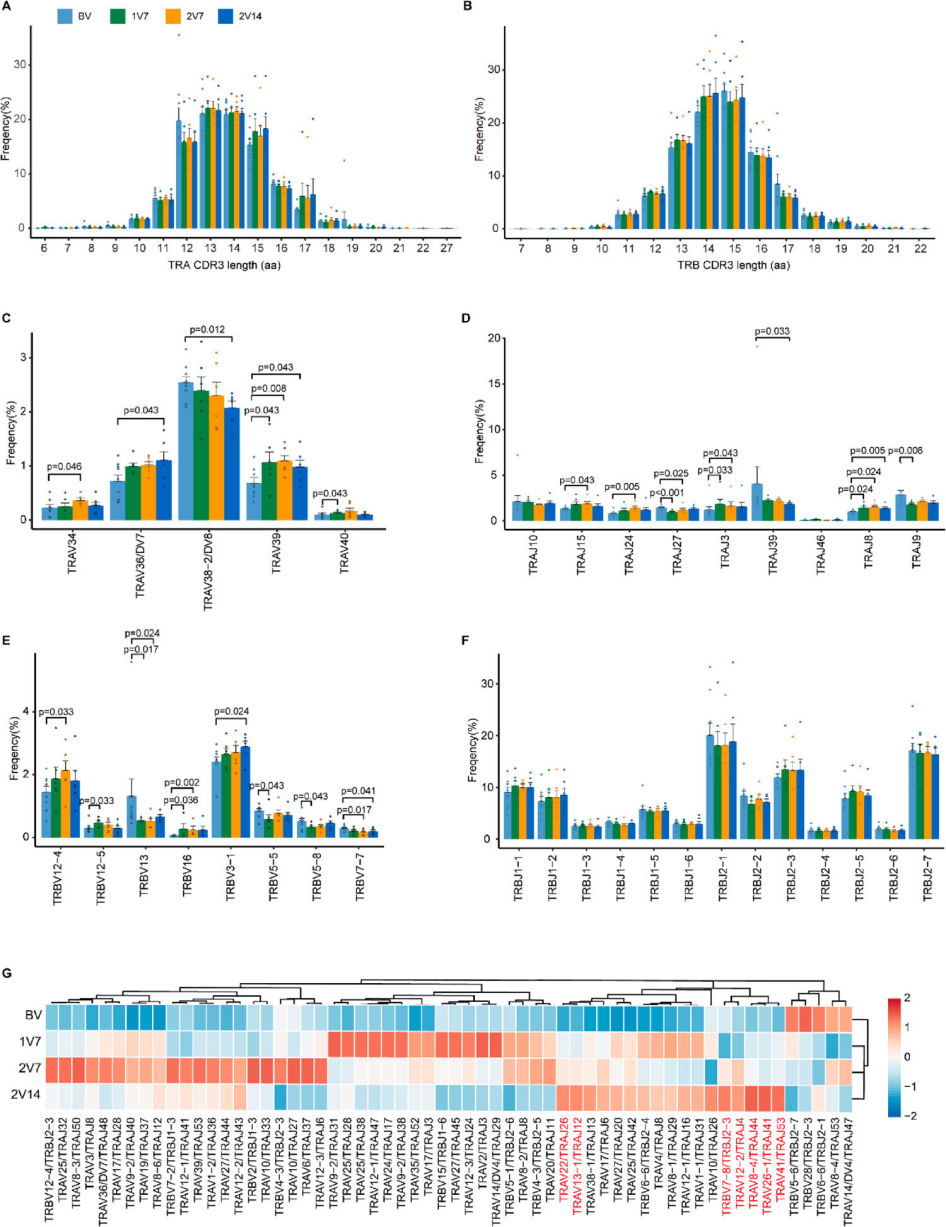
Global profile of the CDR3 length of T cell clones and TCR VJ distribution after BBIBP-CorV vaccination. **A** and **B**. Bar plots showing the distribution of CDR3 amino acid lengths for the TCR α chain (**A**) and TCR β chain (**B**) across the four groups (n = 9 in BV group, n = 6 in the 1V7, 2V7 and 2V14 groups). **C**-**F**. Bar plot showing the usage of some TRAV genes (**C**), TRAJ genes (**D**), TRBV genes (**E**) and TRBJ genes (**F**) across the four groups (n = 9 in BV group, n = 6 in the 1V7, 2V7 and 2V14 groups). Error bars represent ± s.e.m. Data were analyzed by an unpaired Mann-Whitney U-test. A p value< 0.05 was considered statistically significant. **G**. Heatmap showing the usage of significantly differentially-expressed V-J gene pairs of the TCR α and TCR β chains across the four groups. Red, high expression; blue, low expression. CDR3, complementarity determining region 3. BV group, before vaccination. 1V7 group, 7 days after the first vaccination. 2V7 group, 7 days after the second vaccination. 2V14 group, 14 days after the second vaccination.

To gain insight into the dynamics of the TCR repertoire during immune responses to BBIBP-CorV vaccine, we reconstructed full-length TCR sequences from the sequencing data. We first assessed the complementarity determining region 3 (CDR3) length distribution of the TCR α and TCR β chains across four time points. The results showed that the distribution of CDR3 length was not significantly changed before and after vaccination (Figure 4A, B).

We then examined biases in TCR α and TCR β gene usage by comparing repertoire assignments before and after vaccination (Figure 4C-F and S4A). Within the TCR α chain (Figure 4C, D), compared with pre-vaccination, the results showed that: 1) the expression of *TRAV39* and *TRAJ8* was increased in the 1V7, 2V7 and 2V14 groups; 2) the expression of *TRAJ3* was increased in the 1V7 and 2V7 groups and returned to the basal level in the 2V14 group; 3) the expression of *TRAV34*, *TRAJ15* and *TRAJ24* was increased in the 2V7 group and returned to the basal level in the 2V14 group; and 4) the expression of *TRAV36/DV7* was increased in the 2V14 group. Within the TCR β chain (Figure 4E, F), compared with pre-vaccination, the results showed that: 1) the expression of *TRBV16* was increased in the 1V7 and 2V7 groups and returned to the basal level in the 2V14 group; 2) the expression of *TRBV12-5* was increased in the 1V7 group and returned to the basal level in the 2V7 and 2V14 groups; and 3) the expression of *TRBV3-1* was increased in the 2V14 group. In addition, we compared the paired VJ frequencies before and after vaccination (Figure 4G and S4B). The results showed that the frequencies of *TRAV41*/*TRAJ53*, *TRAV26-1*/*TRAJ41* and *TRAV8-4/TRAJ44* were increased in the 2V14 group (Figure 4G and S4C). The selective usage of VJ genes suggested that different immunodominant epitopes may drive the molecular composition of the TCR and may be associated with the BBIBP-CorV-specific T cell response.

### Clonal expansion of CD4^+^ T cells after BBIBP-CorV vaccination

**Figure 5.**
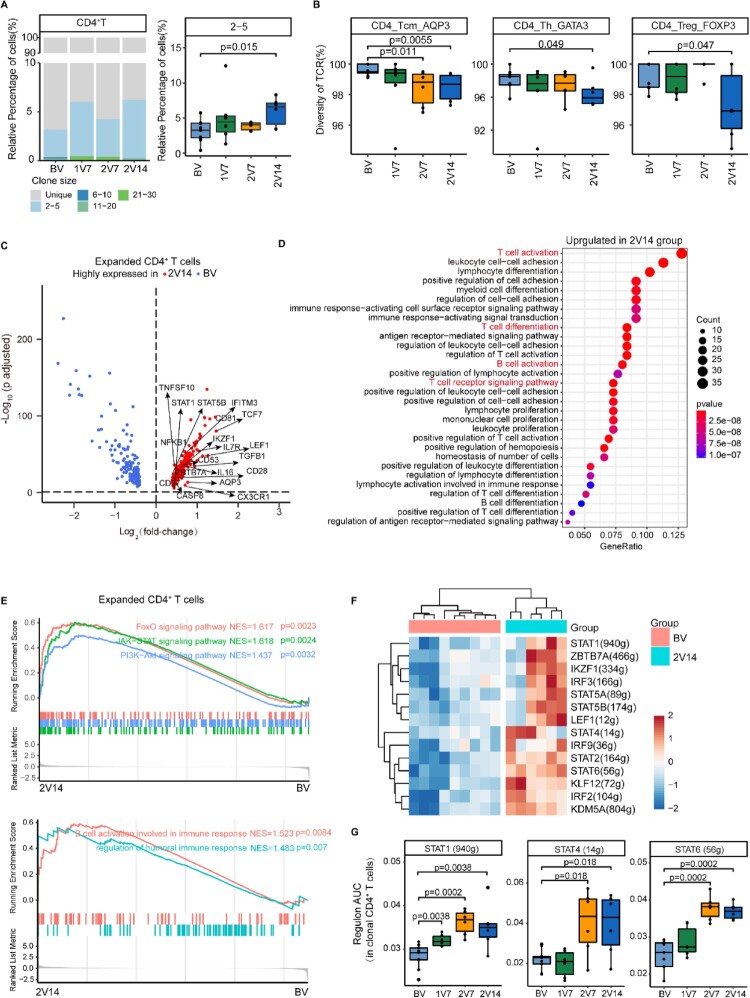
Clonal expansion of CD4+ T cells after BBIBP-CorV vaccination. **A**. Bar plot showing the distribution of CD4^+^ T cell clone sizes across the four groups (left panel). Box plot showing the proportions of CD4^+^ T cells with 2–5 times clonal expansion among the total CD4^+^ T cells across the four groups (n = 9 in BV group, n = 6 in the 1V7, 2V7 and 2V14 groups) (right panel). All box plots display the median, 25th and 75th percentiles, and whiskers extending to the maximum and minimum data points. Data were analyzed by an unpaired Mann-Whitney U-test. A p value< 0.05 was considered statistically significant. **B**. Box plots showing the clone diversity of expanded T cell subsets across the four groups (n = 9 in BV group, n = 6 in the 1V7, 2V7 and 2V14 groups). The percentage of TCR classification count (the number of different TCR sequences in a sample) to the total cell number was used to indicate TCR diversity. All box plots display the median, 25th and 75th percentiles, and whiskers extending to the maximum and minimum data points. Data were analyzed by an unpaired Mann-Whitney U-test. A p value< 0.05 was considered statistically significant. **C**. Volcano plot showing the DEGs in the expanded CD4^+^ T cells (clone size ≥ 2) in the 2V14 group compared with the BV group. Example genes are labelled with gene names. Red, upregulated in the 2V14 group (Log_2_FC ≥ 0.4, adjusted *p* value < 0.05); blue, downregulated in the 2V14 group (Log_2_FC≤−0.4, adjusted *p* value < 0.05). **D**. Functional enrichment analysis of upregulated DEGs in clonal expanded CD4^+^ T cells of the 2V14 group compared with the BV group. The top 30 enriched BP terms are shown. Interesting BP terms are highlighted in red. **E**. GSEA enrichment plots showing five upregulated gene sets in the clonal expanded CD4^+^ T cells of 2V14 group compared with those in the BV group. NES, normalized enrichment score. A p value< 0.05 was statistically significant. **F**. Heatmap showing the inferred significantly differentially-expressed TF regulons between the 2V14 and BV groups. Numbers between brackets represent the regulon counts for respective transcription factors. Red, high expression; blue, low expression. **G**. Box plots showing the area under the curve of STAT1(940 g), STAT4 (14 g) and STAT6 (56 g) in the clonal expanded CD4^+^ T cells derived from BV group (n = 9), 1V7 group (n = 6), 2V7 group (n = 6) and 2V14 group (n = 6). Groups are shown in different colours. Numbers between brackets represent the regulon counts for respective transcription factors. All box plots display the median, 25th and 75th percentiles, and whiskers extending to the maximum and minimum data points. Data were analyzed by an unpaired Mann-Whitney U-test. A p value< 0.05 was considered statistically significant. DEGs, differentially expressed genes. FC, fold change. BP, biological process. BV group, before vaccination. 1V7 group, 7 days after the first vaccination. 2V7 group, 7 days after the second vaccination. 2V14 group, 14 days after the second vaccination.

To further investigate the T cell repertoire and related T cell functions post-BBIBP-CorV vaccination, we analyzed the features of T cell clonal expansion at different time points after vaccination. The percentage of TCR classification counts (the number of different TCR sequences in a sample) to the total cell number was used to indicate TCR diversity. Notably, clonal analysis showed that vaccination induced the expansion of small-sized clones (clone size: 2–5 cells) in CD4^+^ T cells in the 2V14 group (Figure 5A, B); whereas, no obvious clonal expansion was observed in CD8^+^ T cells (Figure S5A). We next calculated the TCR diversity for T cell subsets across four time points (Figure 5B and S5B). Compared with pre-vaccination, the results showed that: 1) the TCR diversity of CD4_Tcm_AQP3 cells was decreased in the 2V7 and 2V14 groups; 2) the TCR diversity of CD4_Th_GATA3, CD4_Treg_FOXP3 and CD8_Naive_LEF1 cells was decreased in the 2V14 group; and 3) the TCR diversity of CD4_Naive_CCR7 cells was decreased in the 1V7 and 2V14 groups (Figure 5B and S5B).

Next, to reveal the functional features of CD4^+^ T cells with expanded TCR clonotypes (cell sizes ≥ 2) between pre- and post-vaccination, we performed DEG analysis on these clonal expanded CD4^+^ T cells, which may represent the most active CD4^+^ T cells against viral antigens. The DEG analysis showed that the expression of Th and memory T cell differentiation transcription factor (TF) genes *TCF7* [42], *LEF1* [43], *STAT1* [44], *STAT5B* [45] and *IKZF1* [46] was upregulated in the 2V7 and 2V14 groups compared with BV group (Figure 5C and S5C). Functional enrichment analysis showed that upregulated DEGs in the 2V7 and 2V14 groups were enriched in “T cell activation”, “T cell differentiation”, “T cell receptor signaling pathway” and “B cell activation” pathways (Figure 5C, D and S5C, D). We next illustrated the properties of these clonal expanded CD4^+^ T cells before and after vaccination by GSEA analysis. FOXO signalling, JAK-STAT signalling, PI3K-Akt signalling, B cell activation and humoral response-related pathways were significantly enriched in these clonal expanded CD4^+^ T cells in the 2V14 group (Figure 5E). To further investigate the transcriptional regulatory features of clonal expanded CD4^+^ T cells after the booster vaccination, we predicted the core TFs using SCENIC (Figure 5F). Consistent with the DEG analysis (Figure 5C), the expression of TF regulons LEF1(12 g), STAT1(940 g), STAT5B(174 g) and IKZF1(334 g) was significantly upregulated in the 2V14 group (Figure 5F). Notably, the expression of TF regulon STAT1(940 g), associated with Th1 cell differentiation, was significantly upregulated after vaccination (1V7, 2V7 and 2V14 groups) (Figure 5G). Whereas, the expression levels of Th1-associated TF regulon STAT4(14 g) and Th2-associated TF regulon STAT6 (56 g) were significantly upregulated in the 2V7 and 2V14 groups (Figure 5G). These results implied a potential role of these TFs in regulating the differentiation of CD4^+^ T cells during the vaccination process.

### Immunological features of B cell subsets after BBIBP-CorV vaccination

**Figure 6.**
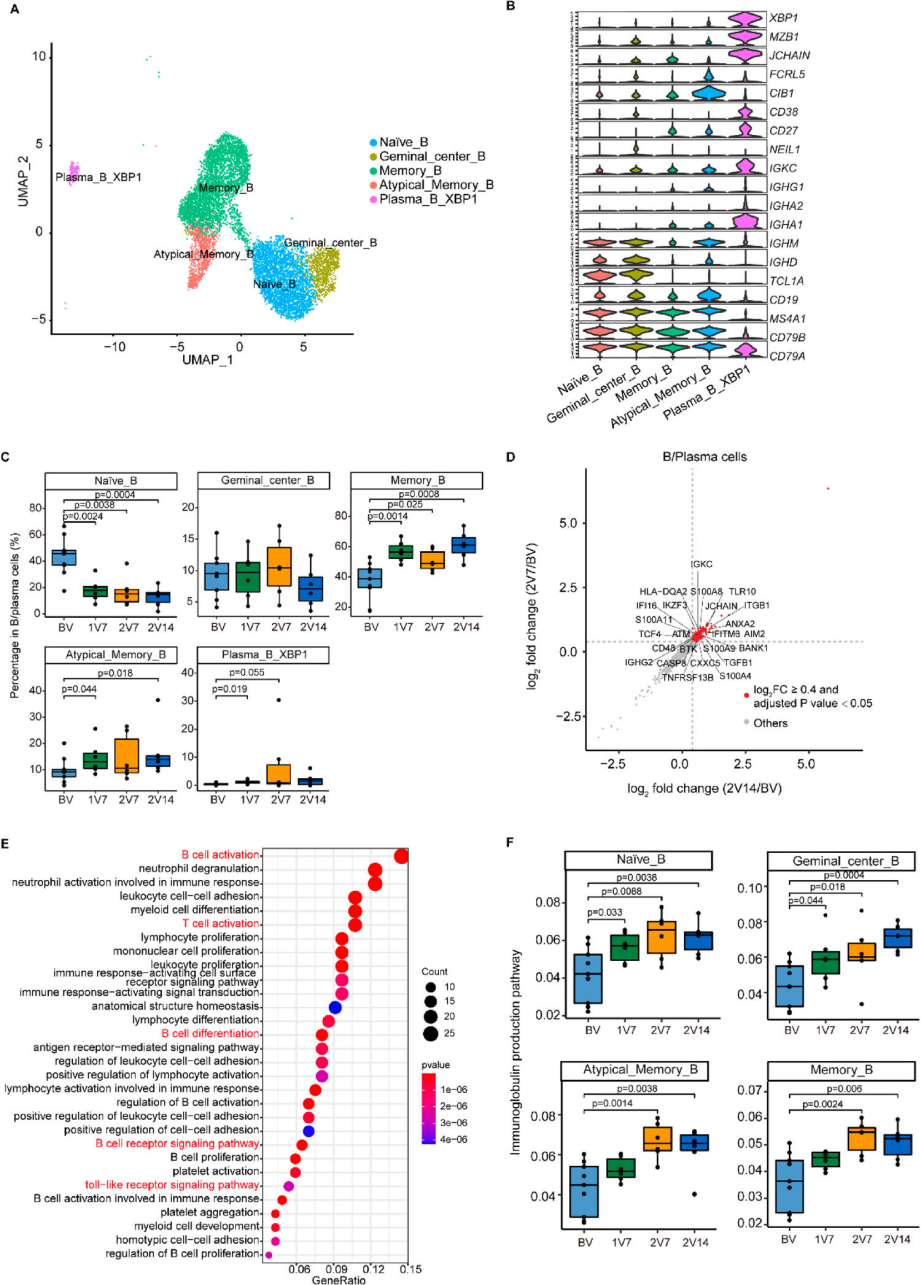
Immunological features of B cell subsets after BBIBP-CorV vaccination. **A**. UMAP plot showing the B/plasma cells coloured according to cell subset. **B**. Violin plots showing expression of signature genes in five B cell subsets. **C**. Box plots showing the proportion of each B cell subset in total B/plasma cells of each sample across four groups (n = 9 in BV group, n = 6 in the 1V7, 2V7 and 2V14 groups). All box plots show the median, 25th and 75th percentiles. Data were analyzed by an unpaired Mann-Whitney U-test. A p value< 0.05 was considered statistically significant. **D**. Scatter-plot showing DEGs in the B/plasma cells of the 2V7 and 2V14 groups compared with those in the BV group. Each red dot denotes an individual gene with Log_2_FC ≥ 0.4 and an adjusted *p* value < 0.05 for the 2V7/BV and 2V14/BV comparisons. **E**. Functional enrichment analysis of the DEGs coloured in red in the scatter-plot in **D**. The top 30 enriched BP terms are shown. Interesting BP terms are highlighted in red. **F**. Box plots showing the expression of the immunoglobulin production pathway across B cell subsets derived from BV group (n = 9), 1V7 group (n = 6), 2V7 group (n = 6) and 2V14 group (n = 6). Groups are shown in different colours. All box plots display the median, 25th and 75th percentiles, and whiskers extending to the maximum and minimum data points. Data were analyzed by an unpaired Mann-Whitney U-test. A p value< 0.05 was considered statistically significant. DEGs, differentially expressed genes. FC, fold change. BP, biological process. BV group, before vaccination. 1V7 group, 7 days after the first vaccination. 2V7 group, 7 days after the second vaccination. 2V14 group, 14 days after the second vaccination.

To trace the dynamic changes in different B/plasma cell subsets, we subclustered B/plasma cells into five subsets according to the expression of B cell signature genes (Figure 6A, B and Supplementary Table 2). These five B/plasma cell subsets included naïve B cells (Naïve_B, *CD79A ^+ ^MS4A1 ^+ ^IGHD^+^*), germinal centre B cells (Germinal_central_B, *CD79A ^+ ^MS4A1 ^+ ^NEIL1^+^*), memory B cells (Memory_B, *MS4A1 ^+ ^CD27^+^*), atypical memory B cells (Atypical_memory_B, *MS4A1 ^+ ^CD27 ^+ ^CIB1^+^*) and plasma B cells (Plasma_B_XBP1, *CD79A ^+ ^CD38 ^+ ^MZB1^+^*) (Figure 6A, B and Supplementary Table 2).

To gain insight into the features of B/plasma cell subsets, we evaluated the distribution of each subset during the vaccination process (Figure 6C). Compared with the BV group, the proportion of memory B (Memory_B) cells was increased after vaccination (1V7, 2V7 and 2V14 groups) (Figure 6C). The proportion of atypical memory B (Atypical_memory_B) cells was increased in the 1V7 and 2V14 groups (Figure 6C). By contrast, a previous scRNA-seq study demonstrated that the proportion of memory B cells was decreased in patients with COVID-19 compared with healthy donors [31]. The proportion of Plasma_B_XBP1 cells was increased in the 1V7 group (Figure 6C). By contrast, the proportion of naïve B cells was decreased after vaccination (1V7, 2V7 and 2V14 groups) (Figure 6C), which was consistent with a recent study on COVID-19 mRNA vaccination involving scRNA-seq analysis [31].

To further investigate differential transcriptomic changes in B/plasma cells after the booster vaccination, we compared the expression profiles of B/plasma cells in the 2V7 and 2V14 groups to those in the BV group (Figure 6D and S6A, B). In comparison with the BV group, functional enrichment analysis showed that the upregulated DEGs in the 2V7 and 2V14 groups were enriched in “B cell activation”, “B cell receptor signaling” and “toll-like receptor signaling pathway” pathways (Figure 6E). Furthermore, we analyzed the expression of immunoglobulin production pathway in B/plasma cell subsets. Compared with the BV group, Naïve_B, Germinal_central_B, Memory_B and Atypical_memory_B cells showed higher enrichment of the immunoglobulin production pathway in the 2V7 and 2V14 groups (Figure 6F).

### Specific rearrangement of the VJ genes of BCR after BBIBP-CorV vaccination

**Figure 7.**
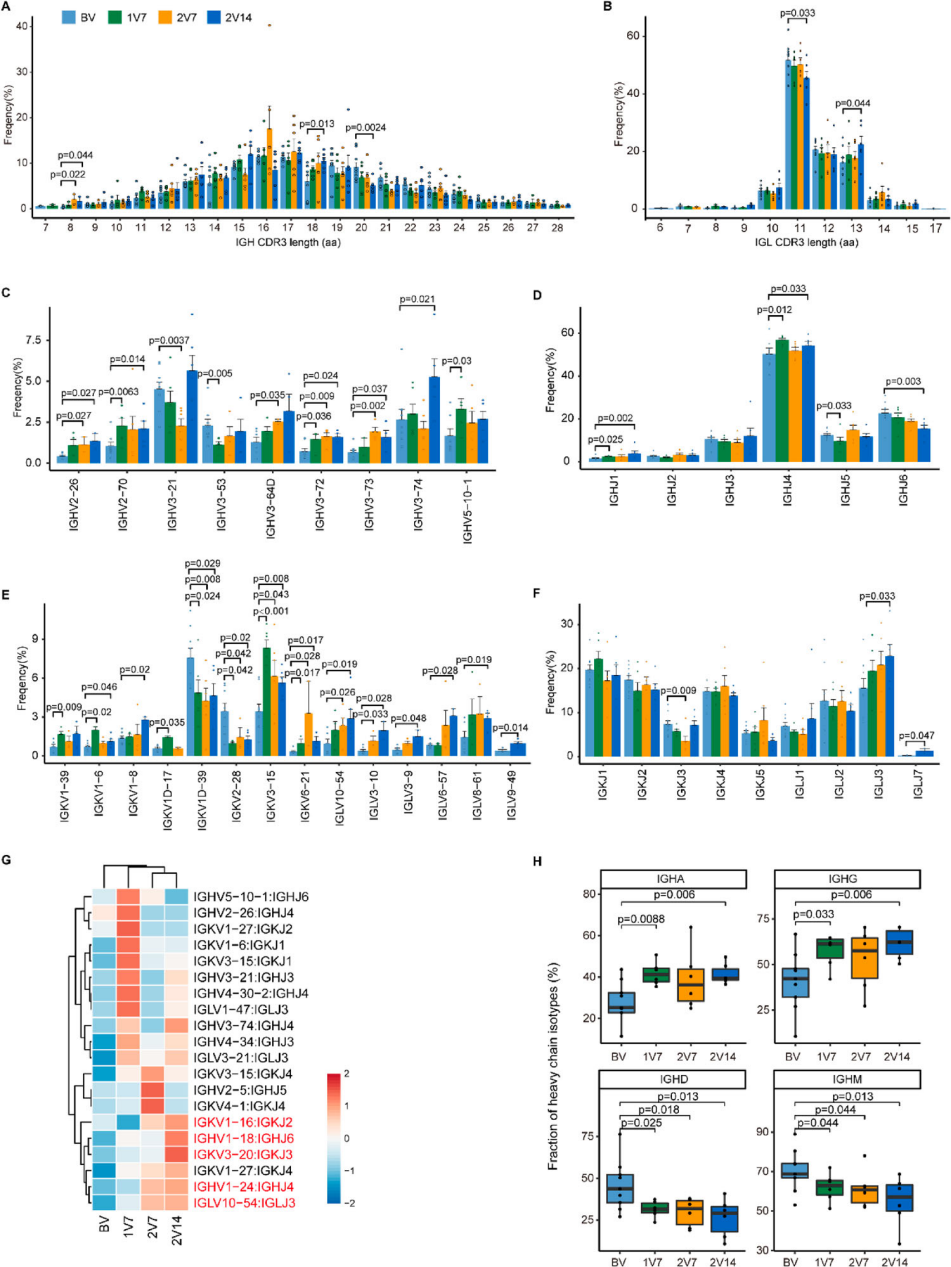
Global profile of the CDR3 length of B cell clones and BCR VJ distribution after BBIBP-CorV vaccination. **A** and **B**. Bar plots showing the distribution of amino acid lengths of CDR3 for the BCR heavy chain (**A**) and BCR light chain (**B**) across the four groups (n = 9 in BV group, n = 6 in the 1V7, 2V7 and 2V14 groups). Error bars represent ± s.e.m. Data were analyzed by an unpaired Mann-Whitney U-test. A p value< 0.05 was considered statistically significant. **C**-**F**. Bar plot showing the usage of IGHV genes (**C**), IGHJ genes (**D**), IGKV/LV genes (**E**) and IGKJ/LJ genes (**F**) across the four groups (n = 9 in BV group, n = 6 in the 1V7, 2V7 and 2V14 groups). Error bars represent ± s.e.m. Data were analyzed by an unpaired Mann–Whitney U-test. A p value< 0.05 was considered statistically significant. **G**. Heatmap showing the usage of significantly differentially-expressed V-J gene pairs of the BCR heavy and light chains across the four groups (n = 9 in BV group, n = 6 in the 1V7, 2V7 and 2V14 groups). Red, high expression; blue, low expression. **H**. Box plots showing the UMI percentages of *IGHD*, *IGHA*, *IGHG* and *IGHM* in B/plasma cells across the four groups (n = 9 in BV group, n = 6 in the 1V7, 2V7 and 2V14 groups). All box plots display the median, 25th and 75th percentiles, and whiskers extending to the maximum and minimum data points. Data were analyzed by an unpaired Mann-Whitney U-test. A p value< 0.05 was considered statistically significant. CDR3, complementarity determining region 3. BV group, before vaccination. 1V7 group, 7 days after the first vaccination. 2V7 group, 7 days after the second vaccination. 2V14 group, 14 days after the second vaccination.

Next, to understand the dynamics of the BCR repertoire during immune responses to BBIBP-CorV vaccine, we reconstructed BCR sequences from BCR sequencing data and analyzed the usage of VJ genes and BCR clonal expansion. We first assessed the length distribution of CDR3 amino acid (aa) sequences across four time points. The results showed that the frequency of 18 aa of heavy chain (Figure 7A) and 13 aa of light chain (Figure 7B) was increased in the 2V14 group compared with the BV group.

We then compared the usage of VJ genes across four time points after vaccination (Figure 7C-F and S7A). Notably, in comparison with the BV group, gene usage analysis showed that: 1) the expression of *IGHV2-26* and *IGHV3-73* was increased in the 2V7 and 2V14 groups (Figure 7C); and 2) the expression of *IGHV3-74* was increased in the 2V14 group. Furthermore, the expression of *IGHJ1* and *IGHJ4* was increased in the 1V7 and 2V14 groups (Figure 7D). In addition, compared with the BV group, we observed that: 1) the expression of *IGKV3-15* was increased after vaccination (1V7, 2V7 and 2V14 groups); 2) the expression of *IGKV3-10* and *IGLV10-54* was increased in the 2V7 and 2V14 groups; and 3) the expression of *IGKV1-8*, *IGLV6-57*, *IGLV8-61* and *IGLJ3* was increased in the 2V14 group (Figure 7E, F). To further study biased V-J rearrangements of the BCR, we compared the paired VJ frequencies across four time points (Figure 7G and S7B). The results showed that: 1) the frequencies of *IGKV1-16/IGKJ2*, *IGHV1-24/IGHJ4* and *IGLV10-54/IGLJ3* were increased in the 2V7 and 2V14 groups; and 2) the frequencies of *IGHV1-18/IGHJ6* and *IGKV3-20/IGKJ3* were increased in the 2V14 group (Figure 7G and S7B).

Then, we evaluated the distribution of immunoglobulin heavy genes, IgA, IgD, IgG and IgM, in B cells across four time points. In comparison with the BV group, the abundance of *IGHA* and *IGHG* was increased in the 1V7 and 2V14 groups (Figure 7H). Whereas, the abundance of *IGHD* and *IGHM* was decreased after vaccination (1V7, 2V7 and 2V14 groups) (Figure 7H). Next, we assessed the clonal BCR expansion status before and after vaccination. The BCR clones of HD1, HD4 and HD5 expanded after the second-dose vaccination in the 2V7 and 2V14 groups (Figure S7C). Taken together, these results revealed the dynamics of the BCR repertoire induced by booster BBIBP-CorV vaccination.

### Comparison of immunological features following BBIBP-CorV vaccination and SARS-CoV-2 infection

**Figure 8.**
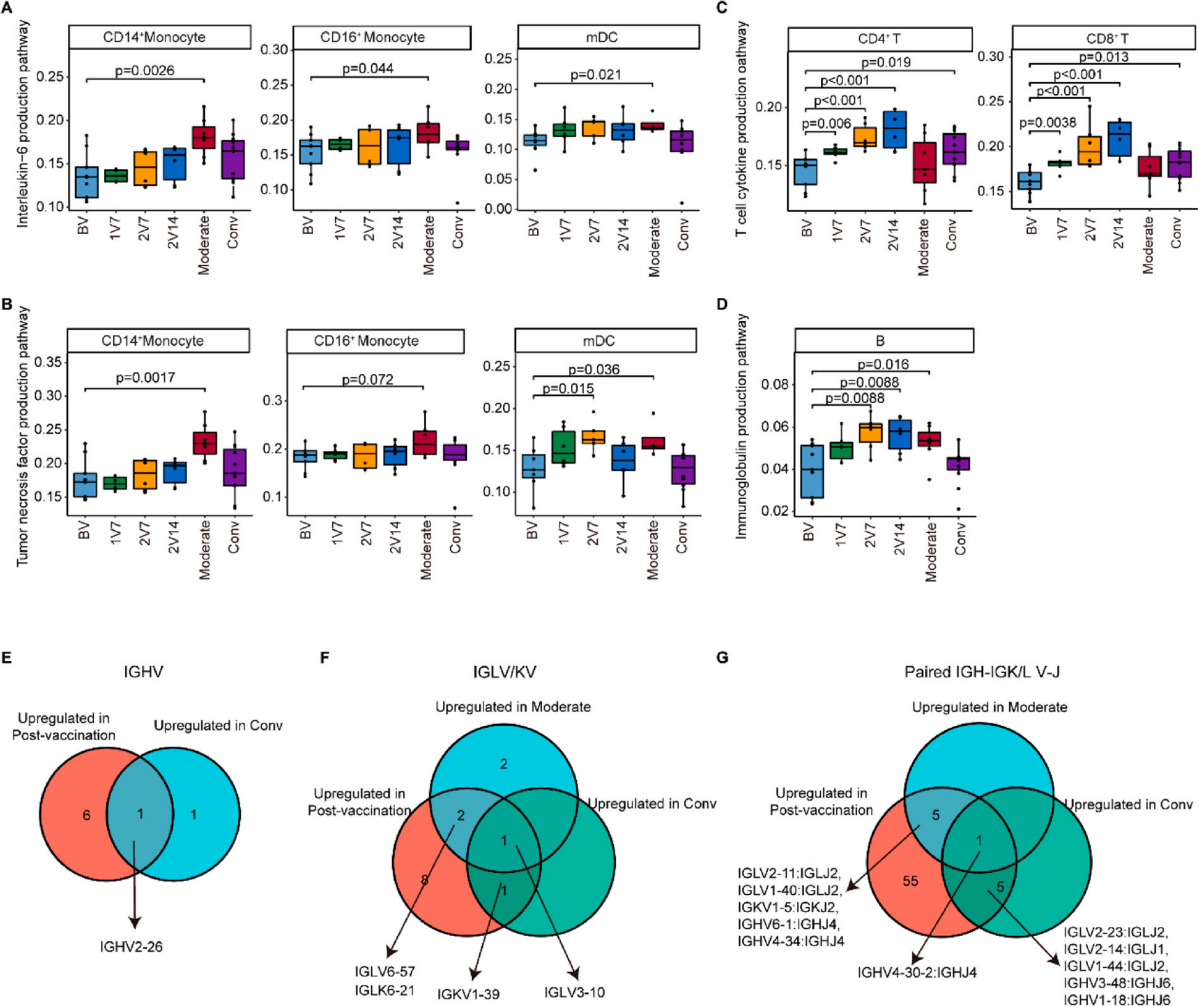
Comparison of the immunological features induced by BBIBP-CorV vaccination and SARS-CoV-2 infection. **A** and **B**. Box plots showing the expression of the interleukin-6 production pathway (**A**) and the tumour necrosis factor production pathway (**B**) in CD14^+^ monocytes, CD16^+^ monocytes and mDCs derived from the BV group (n = 9), 1V7 group (n = 6), 2V7 group (n = 6), 2V14 group (n = 6), moderate group (n = 6) and conv group (n = 11). Groups are shown in different colours. All box plots display the median, 25th and 75th percentiles, and whiskers extending to the maximum and minimum data points. Data were analyzed by an unpaired Mann–Whitney U-test. A p value< 0.05 was considered statistically significant. **C**. Box plots showing the expression of the T cell cytokine production pathway in CD4^+^ and CD8^+^ T cells derived from the BV group (n = 9), 1V7 group (n = 6), 2V7 group (n = 6), 2V14 group (n = 6), moderate group (n = 6) and conv group (n = 11). Groups are shown in different colours. All box plots display the median, 25th and 75th percentiles, and whiskers extending to the maximum and minimum data points. Data were analyzed by an unpaired Mann-Whitney U-test. A p value< 0.05 was considered statistically significant. **D**. Box plots showing the expression of the immunoglobulin production pathway in B cells derived from the BV group (n = 9), 1V7 group (n = 6), 2V7 group (n = 6), 2V14 group (n = 6), moderate group (n = 6) and conv group (n = 11). Groups are shown in different colours. All box plots display the median, 25th and 75th percentiles, and whiskers extending to the maximum and minimum data points. Data were analyzed by an unpaired Mann-Whitney U-test. A p value< 0.05 was considered statistically significant. **E**. Venn diagrams showing the upregulated IGHV gene numbers for the post-vaccination and conv groups compared with the BV group. **F**. Venn diagrams similar to **E**, but for the IGKV/LV gene in the post-vaccination, moderate and conv groups. **G**. Venn diagrams similar to **F**, but for the V-J gene pairs for the BCR heavy and light chains. To be considered upregulated post-vaccination, the genes had to be upregulated in at least one of the three groups (1V7, 2V7 or 2V14). Conv, convalescent patients. BV group, before vaccination. 1V7 group, 7 days after the first vaccination. 2V7 group, 7 days after the second vaccination. 2V14 group, 14 days after the second vaccination.

To reveal the differences in the immune response between vaccination and SARS-CoV-2 infection, we first assessed key pathways that were reported to be associated with symptom development in patients [17] and were also enriched in BBIBP-CorV vaccine recipients (Figure 2D, 2F, 6F and S3C), including the interleukin-6 production pathway, tumour necrosis factor production pathway, type I interferon production pathway, T cell cytokine production pathway and immunoglobulin production pathway. We found that the expression of pro-inflammatory cytokine production-related pathways in monocytes and mDCs (including the IL-6 and TNF-α production pathways) was upregulated in patients with moderate symptoms, whereas no significant changes were observed in individuals following vaccination (Figure 8A, B). The expression of the T cell cytokine production-related genes was upregulated in T cells post-vaccination and in recovered convalescent patients compared with pre-vaccination (Figure 8C), but no significant changes were observed in patients with moderate symptoms (Figure 8C). The expression of the type I interferon production-related genes in monocytes and mDCs was upregulated after the booster vaccination, whereas it increased in the mDCs of patients with moderate symptoms and returned to normal levels in recovered convalescent patients (Figure S8A, B). In addition, compared with pre-vaccination, the expression of the immunoglobulin production pathway in B cells was upregulated after booster vaccination and in patients with moderate symptoms, and returned to normal levels in recovered convalescent patients (Figure 8D). Together, these results revealed the differences and similarities in the immune response, as evidenced by the transcriptome, between vaccination and SARS-CoV-2 infection.

Next, we compared the expression of IGV genes following vaccination or SARS-CoV-2 infection with expression levels pre-vaccination. We found that the expression of *IGHV2-26* increased in vaccinated participants and recovered convalescent patients compared with pre-vaccination participants (Figure 8E). The expression of *IGLV3-10* (Figure 8F) and paired *IGHV4-30-2*/*IGHJ4* (Figure 8G) was upregulated in vaccinated participants, patients with moderate symptoms and recovered convalescent patients. Interestingly, a previous study reported that *IGLV3-10* was involved in the production of N276-dependent HIV-1-neutralizing antibodies [47]. Our results revealed the changes in transcription and the repertoire dynamics of immune cells in response to BBIBP-CorV vaccination and COVID-19 progression.

## Discussion

The establishment of immunity against SARS-CoV-2 by vaccination is a key strategy to reduce the spread and severity of COVID-19. Notably, recent clinical studies demonstrated that the effectiveness of different vaccines, including BBIBP-CorV, mRNA, Ad5-nCoV and CoronaVac, against COVID-19 was distinct [13,14]. Here, we extensively investigated the variability of the single-cell transcriptomes and TCR/BCR repertoires of PBMCs at four time points following vaccination with the inactivated vaccine BBIBP-CorV. We also compared the transcription and repertoire dynamics of immune cells in response to vaccination and SARS-CoV-2 infection. We observed temporally aligned alterations in cell type composition, gene expression and clonal expansion, providing novel insights into BBIBP-CorV-elicited immune responses. Moreover, we identified the similarities and differences in immunity induced by SARS-CoV-2 infection and vaccination.

The adaptive immune response is a major determinant of the clinical outcome following SARS-CoV-2 infection and underpins vaccine efficacy [48]. Notably, there is a direct association between the intensity of a vaccine-elicited T cell response and the capability of the immune system to eliminate virus [49]. Studies have provided evidence that effective prophylactic vaccines against replicating viruses should engage a proper T cell immune response [50]. The distribution analysis of T cell subsets showed significant increases in Th2 cells (CD4_Th_GATA3) and central memory CD4^+^ T (CD4^+^ Tcm) cells (CD4_Tcm_AQP3) after the primary and booster vaccinations. Th2 cells are critical for humoral immunity against extracellular pathogens by producing cytokines such as IL-4 and IL-5 [51]. CD4^+^ Tcm cells, with superior proliferation capacity, are required for long-lasting immunity and are induced by vaccination strategies, including those against influenza [52] and tuberculosis [53]. This is in line with a recent study of Ad5-nCoV vaccination, which demonstrated the expansion of CD4^+^ Tcm cells at day 28 following immunization [19]. Moreover, the TCR diversity of CD4^+^ Tcm cells, Th2 cells and CD4^+^ Treg cells decreased at 2 weeks after the booster vaccination, displaying increased cell numbers and expanded clones, which indicated the rapid response of these T cell types to SARS-Cov-2 infection. CD4^+^ Treg cells are a subset of T cells with the capacity to negatively regulate the immune response, maintaining homeostasis and preventing autoimmunity [54]. TCR specificity is determined through VJ gene rearrangement to generate more efficient clonal amplification, which is essential for a successful immune response [55,56]. In current study, some of highly used VJ genes in post vaccination group have been previously reported to be related to memory CD4 T cell activation. For example, one recent study revealed that *TRAV39* expression was associated with memory CD4 T cell activation in Esophageal Squamous Cell Carcinoma [57]. Clinical evidence showed that deaths from COVID-19 disease were mainly caused by a cytokine storm [58], which is the result of an uncontrolled immune response. Of note, a recent study reported that individuals who received two doses of BBIBP-CorV were protected against the more serious consequences of infection [13]. The small clone expansion of CD4^+^ Treg cells might be involved in controlling the overactive immune response and cytokine storm induced by SARS-CoV-2 in severe cases, thus BBIBP-CorV vaccination reduces the risk of serious disease and death.

The presence of neutralizing antibodies is currently used as a surrogate indicator of immunity. Our data indicated that neutralizing antibody levels were significantly higher in the vaccinated groups. In addition, the number of memory B cells increased following the booster vaccination. Consistent with our results, recent studies using ELISA and flow cytometry also demonstrated that BBIBP-CorV vaccine induced neutralizing antibody production and spike- or RBD-specific memory B cells following the standard two-dose vaccination regimen [26,59]. A high level of IgG effectively protects against virus infection [60], while IgA is the major mucosal antibody that constitutes an important first line of defense [61]. Compared with pre-vaccination, the abundance of *IGHG* and *IGHA* was increased after the booster vaccination, indicating the high efficacy of the B cell response following two doses of BBIBP-Cov2 vaccine.

Additionally, we found that there was an increase in BCR clonal expansion and skewed usage of VJ genes of BCR in the vaccinated individuals. Notably, some of those biased usage of VJ genes were reported to be associated with SARS-CoV-2-specific immune responses and antibodies. For example, *IGLV3-10* and *IGHV1-18*/*IGHJ6* are involved in the human B cell response to SARS-CoV-2 virus [62,63], and *IGHV2-70* and paired *IGHV1-24*/*IGHJ4* are involved in the production of anti–SARS-CoV-2 neutralizing antibodies [64,65]. Collectively, these data may help pave the way for the development of the next-generation precision vaccine.

Comparison of the immune responses elicited by SARS-CoV-2 infection and vaccination will help us to better understand how BBIBP-CorV protects against the risk of serious disease and death. The expression of genes associated with the production of pro-inflammatory cytokines, such as IL-6 and TNF-α, did not change significantly in BBIBP-CorV vaccination recipients, despite these genes being significantly upregulated in patients with moderate symptoms. The systemic distribution of TNF-α and IL-6 is a major trigger of the cytokine storm observed in some patients [66], as well as some of the side effects induced by the vaccine [67]. Moreover, CD8^+^ T cells are the mainly clonal expanded population of cells in patients recovering from SARS-CoV-2 infection [68] and those vaccinated with Ad5-nCoV [19]. Additionally, mRNA vaccines, including BNT162b2 and mRNA-1273, were shown to elicit CD8^+^ T cells clonal expansion and the Th1 cell responses without affecting Th2 cell responses [69]. However, our analysis of TCR repertoires revealed that CD4^+^ T cells (but not CD8^+^ T cells) displayed an expansion following BBIBP-CorV vaccination. These data suggested that the inactivated vaccines mainly induce a Th2-B cell immune response favouring the production of anti-SARS-Cov-2 antibodies [70].

Clinical studies of two types of inactivated SARS-CoV-2 vaccines, BBIBP-CorV and CoronaVac, showed that BBIBP-CorV exhibits a higher protection rate against severe cases and a lower infection rate with SARS-Cov-2 compared with CoronaVac [13]. A recent scRNA-seq study revealed that inactivated vaccine CoronaVac downregulated anti-viral signalling pathways (i.e. type I interferon responses) and elevated pro-inflammatory signalling pathways (i.e. NF-κB signalling) [21], which was associated with side effects and systemic inflammation. However, the functional transcription and TCR/BCR repertoire dynamics of immune cells had not been defined.

Although a recent scRNA-seq study indicated that BBIBP-CorV could induce an efficient humoral immune response, the cellular immune response, which is the pivot of humoral immune response, remained to be further investigated [71]. Our current study indicated that the inactivated vaccine BBIBP-CorV activated an anti-viral immune response with clonal expansion of Th2 cells, central memory CD4^+^ T cells and Treg cells. The distinct immune features induced by these two SARS-CoV-2 inactivated vaccines may contribute to the different protection rates against severe cases of SARS-CoV-2 infection.

Based on the abovementioned findings, we hypothesize that: 1) inflammatory monocytes induced by booster vaccination optimize immune mobilization against SASR-CoV-2 infection; 2) the Th2 cells and CD4^+^ Tcm cells induced by booster vaccination rapidly respond to SASR-CoV-2 infection, thereby preventing disease exacerbation; 3) post-booster vaccination, memory B cells rapidly produce neutralizing antibody with the help of CD4^+^ T cells; and 4) along with the immune response against SASR-CoV-2, expanded CD4^+^ Treg clones act as a brake, preventing an excessive immune response induced by inflammatory immune cells, and orchestrating appropriate immunity against SASR-CoV-2 in the vaccination recipients.

Taken together, our study provides novel insights into the cellular and molecular basis of the post-SARS-CoV-2 vaccination response at single-cell resolution, especially the CD4^+^ T cell-dependent antibody response induced by BBIBP-CorV. Our findings may facilitate the development of more potent, durable and safe prophylactic vaccines against SARS-CoV-2 infection. However, the present study is limited by its small sample size. Further studies involving a larger number of samples and longer follow-up times are required to understand the long-term cellular and humoral immune responses to SARS-CoV-2 vaccines. In addition, the combination of single-cell RNA and TCR/BCR sequencing analysis revealed that the BBIBP-CorV vaccination might induce antigen-specific expansion of T and B cells, while further studies with more direct evidence are needed to validate these observations.

## Supplementary Material

Supplemental Material

## References

[1] Lu S. Timely development of vaccines against SARS-CoV-2. Emerg Microbes Infect. 2020;9(1):542–544. 32148172 10.1080/22221751.2020.1737580 PMC7144304

[2] Xia S, Zhang Y, Wang Y, et al. Safety and immunogenicity of an inactivated SARS-CoV-2 vaccine, BBIBP-CorV: a randomised, double-blind, placebo-controlled, phase 1/2 trial. Lancet Infect Dis. 2021 Jan;21(1):39–51. 33069281 10.1016/S1473-3099(20)30831-8 PMC7561304

[3] Ai J, Zhang Y, Zhang H, et al. Safety and immunogenicity of a third-dose homologous BBIBP-CorV boosting vaccination: interim results from a prospective open-label study. Emerg Microbes Infect. 2022;11(1):639–647. 35034582 10.1080/22221751.2022.2025746 PMC8881062

[4] Wu Z, Hu Y, Xu M, et al. Safety, tolerability, and immunogenicity of an inactivated SARS-CoV-2 vaccine (CoronaVac) in healthy adults aged 60 years and older: a randomised, double-blind, placebo-controlled, phase 1/2 clinical trial. Lancet Infect Dis. 2021 Jun;21(6):803–812. 33548194 10.1016/S1473-3099(20)30987-7 PMC7906628

[5] Polack FP, Thomas SJ, Kitchin N, et al. Safety and efficacy of the BNT162b2 mRNA COVID-19 vaccine. N Engl J Med. 2020 Dec 31;383(27):2603–2615. 33301246 10.1056/NEJMoa2034577 PMC7745181

[6] Baden LR, El Sahly HM, Essink B, et al. Efficacy and safety of the mRNA-1273 SARS-CoV-2 vaccine. N Engl J Med. 2021 Feb 4;384(5):403–416. 33378609 10.1056/NEJMoa2035389 PMC7787219

[7] Voysey M, Clemens SAC, Madhi SA, et al. Safety and efficacy of the ChAdOx1 nCoV-19 vaccine (AZD1222) against SARS-CoV-2: an interim analysis of four randomised controlled trials in Brazil, South Africa, and the UK. Lancet. 2021 Jan 9;397(10269):99–111. 33306989 10.1016/S0140-6736(20)32661-1 PMC7723445

[8] Logunov DY, Dolzhikova IV, Shcheblyakov DV, et al. Safety and efficacy of an rAd26 and rAd5 vector-based heterologous prime-boost COVID-19 vaccine: an interim analysis of a randomised controlled phase 3 trial in Russia. Lancet. 2021 Feb 20;397(10275):671–681. 33545094 10.1016/S0140-6736(21)00234-8 PMC7852454

[9] Keech C, Albert G, Cho I, et al. Phase 1-2 trial of a SARS-CoV-2 recombinant spike protein nanoparticle vaccine. N Engl J Med. 2020 Dec 10;383(24):2320–2332. 32877576 10.1056/NEJMoa2026920 PMC7494251

[10] Dai L, Gao L, Tao L, et al. Efficacy and safety of the RBD-dimer-based COVID-19 vaccine ZF2001 in adults. N Engl J Med. 2022 May 4. 10.1056/NEJMoa2202261 PMC9127771 35507481

[11] Kreier F. Ten billion COVID vaccinations: world hits new milestone. Nature. 2022 Jan 31. doi: 10.1038/d41586-022-00285--2. Online ahead of print. 35102290

[12] Ranzani OT, Hitchings MDT, Dorion M, et al. Effectiveness of the CoronaVac vaccine in older adults during a gamma variant associated epidemic of COVID-19 in Brazil: test negative case-control study. Br Med J. 2021 Aug 20;374:n2015. 34417194 10.1136/bmj.n2015 PMC8377801

[13] Premikha M, Chiew CJ, Wei WE, et al. Comparative effectiveness of mRNA and inactivated whole virus vaccines against COVID-19 infection and severe disease in Singapore. Clin Infect Dis. 2022 Oct 12;75(8):1442–1445. 10.1093/cid/ciac288 PMC9047219 35412612

[14] Acevedo ML, Gaete-Argel A, Alonso-Palomares L, et al. Differential neutralizing antibody responses elicited by CoronaVac and BNT162b2 against SARS-CoV-2 lambda in Chile. Nat Microbiol. 2022 Apr;7(4):524–529. 35365787 10.1038/s41564-022-01092-1

[15] Yan VKC, Wan EYF, Ye X, et al. Effectiveness of BNT162b2 and CoronaVac vaccinations against mortality and severe complications after SARS-CoV-2 Omicron BA.2 infection: a case–control study. Emerg Microbes Infect. 2022;11(1):2304–2314. 35980089 10.1080/22221751.2022.2114854 PMC9553171

[16] Papalexi E, Satija R. Single-cell RNA sequencing to explore immune cell heterogeneity. Nat Rev Immunol. 2018 Jan;18(1):35–45. 28787399 10.1038/nri.2017.76

[17] Ren X, Wen W, Fan X, et al. COVID-19 immune features revealed by a large-scale single-cell transcriptome atlas. Cell. 2021 Apr 1;184(7):1895–1913.e19. 33657410 10.1016/j.cell.2021.01.053 PMC7857060

[18] Arunachalam PS, Scott MKD, Hagan T, et al. Systems vaccinology of the BNT162b2 mRNA vaccine in humans. Nature. 2021 Aug;596(7872):410–416. 34252919 10.1038/s41586-021-03791-x PMC8761119

[19] Cao Q, Wu S, Xiao C, et al. Integrated single-cell analysis revealed immune dynamics during Ad5-nCoV immunization. Cell Discov. 2021 Aug 10;7(1):64. 34373443 10.1038/s41421-021-00300-2 PMC8352953

[20] Xie T, Lu S, He Z, et al. Three doses of prototypic SARS-CoV-2 inactivated vaccine induce cross-protection against its variants of concern. Signal Transduct Target Ther. 2022 Feb 25;7(1):61. 35217639 10.1038/s41392-022-00920-4 PMC8873345

[21] Liu J, Wang J, Xu J, et al. Comprehensive investigations revealed consistent pathophysiological alterations after vaccination with COVID-19 vaccines. Cell Discov. 2021 Oct 26;7(1):99. 34697287 10.1038/s41421-021-00329-3 PMC8546144

[22] World medical association declaration of Helsinki: ethical principles for medical research involving human subjects. Jama. 2013 Nov 27;310(20):2191–2194. 24141714 10.1001/jama.2013.281053

[23] Corkum CP, Ings DP, Burgess C, et al. Immune cell subsets and their gene expression profiles from human PBMC isolated by vacutainer cell preparation tube (CPT™) and standard density gradient. BMC Immunol. 2015 Aug 26;16:48. 26307036 10.1186/s12865-015-0113-0 PMC4549105

[24] Zhang H, Liu X, Liu Q, et al. Serological reactivity of inactivated SARS-CoV-2 vaccine based on an S-RBD neutralizing antibody assay. Int J Infect Dis. 2022 Apr;117:169–173. 35121124 10.1016/j.ijid.2022.01.064 PMC8806397

[25] Shi J, Tong R, Zhou M, et al. Circadian nuclear receptor Rev-erbα is expressed by platelets and potentiates platelet activation and thrombus formation. Eur Heart J. 2022 Jun 21;43(24):2317–2334. 35267019 10.1093/eurheartj/ehac109 PMC9209009

[26] Zhang H, Liu Y, Liu D, et al. Time of day influences immune response to an inactivated vaccine against SARS-CoV-2. Cell Res. 2021 Nov;31(11):1215–1217. 34341489 10.1038/s41422-021-00541-6 PMC8326654

[27] Kapellos TS, Bonaguro L, Gemünd I, et al. Human monocyte subsets and phenotypes in major chronic inflammatory diseases. Front Immunol. 2019;10:2035. 31543877 10.3389/fimmu.2019.02035 PMC6728754

[28] Lee S, Ryu JH. Influenza viruses: innate immunity and mRNA vaccines. Front Immunol. 2021;12:710647. 34531860 10.3389/fimmu.2021.710647 PMC8438292

[29] Fujimura K, Oyamada A, Iwamoto Y, et al. CD4 t cell-intrinsic IL-2 signaling differentially affects Th1 and Th17 development. J Leukoc Biol. 2013 Aug;94(2):271–279. 23715742 10.1189/jlb.1112581

[30] Li-Weber M, Krammer PH. Regulation of IL4 gene expression by T cells and therapeutic perspectives. Nat Rev Immunol. 2003 Jul;3(7):534–543. 12876556 10.1038/nri1128

[31] Sureshchandra S, Lewis SA, Doratt BM, et al. Single-cell profiling of T and B cell repertoires following SARS-CoV-2 mRNA vaccine. JCI Insight. 2021 Dec 22;6(24). 10.1172/jci.insight.153201 PMC8783687 34935643

[32] Kayamuro H, Yoshioka Y, Abe Y, et al. Interleukin-1 family cytokines as mucosal vaccine adjuvants for induction of protective immunity against influenza virus. J Virol. 2010 Dec;84(24):12703–12712. 20881038 10.1128/JVI.01182-10 PMC3004317

[33] Zhong C, Liu F, Hajnik RJ, et al. Type I interferon promotes humoral immunity in viral vector vaccination. J Virol. 2021 Oct 27;95(22):e0092521. 34495698 10.1128/JVI.00925-21 PMC8549508

[34] Zhang JY, Wang XM, Xing X, et al. Single-cell landscape of immunological responses in patients with COVID-19. Nat Immunol. 2020 Sep;21(9):1107–1118. 32788748 10.1038/s41590-020-0762-x

[35] Nakayama T, Hirahara K, Onodera A, et al. Th2 cells in health and disease. Annu Rev Immunol. 2017 Apr 26;35:53–84. 27912316 10.1146/annurev-immunol-051116-052350

[36] Walker JA, McKenzie ANJ. T(H)2 cell development and function. Nat Rev Immunol. 2018 Feb;18(2):121–133. 29082915 10.1038/nri.2017.118

[37] Raphael I, Joern RR, Forsthuber TG. Memory CD4(+) T cells in immunity and autoimmune diseases. Cells. 2020 Feb 25;9(3). 10.3390/cells9030531 PMC7140455 32106536

[38] Hur JY, Frost GR, Wu X, et al. The innate immunity protein IFITM3 modulates γ-secretase in Alzheimer's disease. Nature. 2020 Oct;586(7831):735–740. 32879487 10.1038/s41586-020-2681-2 PMC7919141

[39] Li M, Zhang D, Zhu M, et al. Roles of SAMHD1 in antiviral defense, autoimmunity and cancer. Rev Med Virol. 2017 Jul;27(4). 10.1002/rmv.1931 28444859

[40] Pizzolato G, Kaminski H, Tosolini M, et al. Single-cell RNA sequencing unveils the shared and the distinct cytotoxic hallmarks of human TCRVδ1 and TCRVδ2 γδ T lymphocytes. Proc Natl Acad Sci U S A. 2019 Jun 11;116(24):11906–11915. 31118283 10.1073/pnas.1818488116 PMC6576116

[41] Bongen E, Vallania F, Utz PJ, et al. KLRD1-expressing natural killer cells predict influenza susceptibility. Genome Med. 2018 Jun 14;10(1):45. 29898768 10.1186/s13073-018-0554-1 PMC6001128

[42] Escobar G, Mangani D, Anderson AC. T cell factor 1: A master regulator of the T cell response in disease. Sci Immunol. 2020 Nov 6;5(53). 10.1126/sciimmunol.abb9726 PMC8221367 33158974

[43] Choi YS, Gullicksrud JA, Xing S, et al. LEF-1 and TCF-1 orchestrate T(FH) differentiation by regulating differentiation circuits upstream of the transcriptional repressor Bcl6. Nat Immunol. 2015 Sep;16(9):980–990. 26214741 10.1038/ni.3226 PMC4545301

[44] Mikhak Z, Fleming CM, Medoff BD, et al. STAT1 in peripheral tissue differentially regulates homing of antigen-specific Th1 and Th2 cells. J Immunol. 2006 Apr 15;176(8):4959–4967. 16585592 10.4049/jimmunol.176.8.4959

[45] Majri SS, Fritz JM, Villarino AV, et al. STAT5B: a differential regulator of the life and death of CD4(+) effector memory T cells. J Immunol. 2018 Jan 1;200(1):110–118. 29187589 10.4049/jimmunol.1701133 PMC5736408

[46] Powell MD, Read KA, Sreekumar BK, et al. Ikaros zinc finger transcription factors: regulators of cytokine signaling pathways and CD4(+) T helper cell differentiation. Front Immunol. 2019;10:1299. 31244845 10.3389/fimmu.2019.01299 PMC6563078

[47] Wibmer CK, Gorman J, Anthony CS, et al. Structure of an N276-dependent HIV-1 neutralizing antibody targeting a rare V5 glycan hole adjacent to the CD4 binding site. J Virol. 2016 Nov 15;90(22):10220–10235. 27581986 10.1128/JVI.01357-16 PMC5105658

[48] Moss P. The T cell immune response against SARS-CoV-2. Nat Immunol. 2022 Feb;23(2):186–193. 35105982 10.1038/s41590-021-01122-w

[49] Saad-Roy CM, Wagner CE, Baker RE, et al. Immune life history, vaccination, and the dynamics of SARS-CoV-2 over the next 5 years. Science. 2020;370(6518):811–818. 32958581 10.1126/science.abd7343 PMC7857410

[50] Hansen SG, Ford JC, Lewis MS, et al. Profound early control of highly pathogenic SIV by an effector memory T-cell vaccine. Nature. 2011 May 26;473(7348):523–527. 21562493 10.1038/nature10003 PMC3102768

[51] Ruterbusch M, Pruner KB, Shehata L, et al. In vivo CD4(+) T cell differentiation and function: revisiting the Th1/Th2 paradigm. Annu Rev Immunol. 2020 Apr 26;38:705–725. 32340571 10.1146/annurev-immunol-103019-085803

[52] Wilkinson TM, Li CK, Chui CS, et al. Preexisting influenza-specific CD4+ T cells correlate with disease protection against influenza challenge in humans. Nat Med. 2012 Jan 29;18(2):274–280. 22286307 10.1038/nm.2612

[53] White AD, Sarfas C, Sibley LS, et al. Protective efficacy of inhaled BCG vaccination against ultra-Low dose aerosol M. tuberculosis challenge in rhesus macaques. Pharmaceutics. 2020 Apr 25;12(5). 10.3390/pharmaceutics12050394 PMC7284565 32344890

[54] Sakaguchi S, Mikami N, Wing JB, et al. Regulatory T cells and human disease. Annu Rev Immunol. 2020 Apr 26;38:541–566. 32017635 10.1146/annurev-immunol-042718-041717

[55] Song C, Pan W, Brown B, et al. Immune repertoire analysis of normal Chinese donors at different ages. Cell Prolif. 2022 Nov;55(11):e13311. 35929064 10.1111/cpr.13311 PMC9628227

[56] Patil VS, Madrigal A, Schmiedel BJ, et al. Precursors of human CD4(+) cytotoxic T lymphocytes identified by single-cell transcriptome analysis. Sci Immunol. 2018 Jan 19;3(19). 10.1126/sciimmunol.aan8664 PMC5931334 29352091

[57] Xu J, Tang L, Wang Z, et al. MIR548P and TRAV39 are potential indicators of tumor microenvironment and novel prognostic biomarkers of esophageal squamous cell carcinoma. J Oncol. 2022;2022:3152114. 36164348 10.1155/2022/3152114 PMC9509226

[58] Ramasamy S, Subbian S. Critical determinants of cytokine storm and type I interferon response in COVID-19 pathogenesis. Clin Microbiol Rev. 2021 Jun 16;34(3). 10.1128/CMR.00299-20 PMC8142516 33980688

[59] Liu Y, Zeng Q, Deng C, et al. Robust induction of B cell and T cell responses by a third dose of inactivated SARS-CoV-2 vaccine. Cell Discov. 2022 Feb 1;8(1):10. 35102140 10.1038/s41421-022-00373-7 PMC8803973

[60] Rockman S, Lowther S, Camuglia S, et al. Intravenous immunoglobulin protects against severe pandemic influenza infection. EBioMedicine. 2017 May;19:119–127. 28408242 10.1016/j.ebiom.2017.04.010 PMC5440604

[61] Woof JM, Kerr MA. The function of immunoglobulin A in immunity. J Pathol. 2006 Jan;208(2):270–282. 16362985 10.1002/path.1877

[62] Leng L, Li M, Li W, et al. Sera proteomic features of active and recovered COVID-19 patients: potential diagnostic and prognostic biomarkers. Signal Transduct Target Ther. 2021 Jun 3;6(1):216. 34083512 10.1038/s41392-021-00612-5 PMC8173321

[63] Zhang Y, Yan Q, Luo K, et al. Analysis of B cell receptor repertoires reveals key signatures of the systemic B cell response after SARS-CoV-2 infection. J Virol. 2022 Feb 23;96(4):e0160021. 34878902 10.1128/jvi.01600-21 PMC8865482

[64] Kim C, Ryu DK, Lee J, et al. A therapeutic neutralizing antibody targeting receptor binding domain of SARS-CoV-2 spike protein. Nat Commun. 2021 Jan 12;12(1):288. 33436577 10.1038/s41467-020-20602-5 PMC7803729

[65] Andreano E, Nicastri E, Paciello I, et al. Extremely potent human monoclonal antibodies from COVID-19 convalescent patients. Cell. 2021 Apr 1;184(7):1821–1835.e16. 33667349 10.1016/j.cell.2021.02.035 PMC7901298

[66] Ragab D, Salah Eldin H, Taeimah M, et al. The COVID-19 cytokine storm; what we know so far. Front Immunol. 2020;11:1446. 32612617 10.3389/fimmu.2020.01446 PMC7308649

[67] Christian LM, Porter K, Karlsson E, et al. Proinflammatory cytokine responses correspond with subjective side effects after influenza virus vaccination. Vaccine. 2015 Jun 26;33(29):3360–3366. 26027906 10.1016/j.vaccine.2015.05.008 PMC4467994

[68] Luo L, Liang W, Pang J, et al. Dynamics of TCR repertoire and T cell function in COVID-19 convalescent individuals. Cell Discov. 2021 Sep 28;7(1):89. 34580278 10.1038/s41421-021-00321-x PMC8476510

[69] Sadarangani M, Marchant A, Kollmann TR. Immunological mechanisms of vaccine-induced protection against COVID-19 in humans. Nat Rev Immunol. 2021 Aug;21(8):475–484. 34211186 10.1038/s41577-021-00578-z PMC8246128

[70] Blum JS, Wearsch PA, Cresswell P. Pathways of antigen processing. Annu Rev Immunol. 2013;31:443–473. 23298205 10.1146/annurev-immunol-032712-095910 PMC4026165

[71] Ai J, Guo J, Zhang H, et al. Cellular basis of enhanced humoral immunity to SARS-CoV-2 upon homologous or heterologous booster vaccination analyzed by single-cell immune profiling. Cell Discov. 2022 Oct 21;8(1):114. 36270988 10.1038/s41421-022-00480-5 PMC9587260

