# Supplementary material for: Characterizing the cellular and molecular variabilities of peripheral immune cells in healthy recipients of BBIBP-CorV inactivated SARS-CoV-2 vaccine by single-cell RNA sequencing
Source: Emerg Microbes Infect. 2023 May 9;12(1):e2187245. doi: 10.1080/22221751.2023.2187245 (PMC10171127; doi:10.1080/22221751.2023.2187245)
Supplement: Supplemental Material [file TEMI_A_2187245_SM3392.zip › Supplementary Figures and Tables List.docx]

**Characterizing cellular and molecular variabilities of peripheral immune cells in healthy** **recipients of BBIBP-CorV** **inactivated SARS-CoV-2 vaccine by single-cell RNA sequencing**

Renyang Tong^1#^, Lingjie Luo^2#^, Yichao Zhao^1#^, Mingze Sun^1#^, Ronghong Li^1^, Jianmei Zhong^1^, Yifan Chen^1^, Liuhua Hu^1^, Zheng Li^1^, Jianfeng Shi^1^, Yuyan Lyu^1^, Li Hu^1^, Xiao Guo^1^, Qi Liu^1^, Tian Shuang^1^, Chenjie Zhang^1^, Ancai Yuan^1^, Lingyue Sun^1^, Zheng Zhang^3^, Kun Qian^1,4^, Lei Chen^1^, Wei Lin^1^, Alex F Chen^5^, Feng Wang^2*^, Jun Pu^1*^

^1^ Division of Cardiology, Shanghai Immune Therapy Institute, Renji Hospital, School of Medicine, Shanghai Jiao Tong University, Shanghai, China

^2^ Shanghai Institute of Immunology, Department of Immunology and Microbiology, State Key Laboratory of Oncogenes and Related Genes, School of Medicine, Shanghai Jiao Tong University, Shanghai, China

^3^ Institute for Hepatology, National Clinical Research Center for Infectious Disease, Shenzhen Third People’s Hospital; The Second Affiliated Hospital, School of Medicine, Southern University of Science and Technology, Shenzhen, Guangdong, China

^4^ School of Biomedical Engineering, and Med-X Research Institute, Shanghai Jiao Tong University, Shanghai, China

^5^ Institute for Developmental and Regenerative Cardiovascular Medicine, Xinhua Hospital, Shanghai Jiao Tong University School of Medicine, Shanghai, China

^*^ To whom correspondence should be addressed:

Jun Pu, MD, Ph.D.; State Key Laboratory for Oncogenes and Related Genes, Division of Cardiology, School of Medicine, Renji Hospital, Shanghai Cancer Institute, Shanghai Jiao Tong University, Shanghai, China. E-mail: [pujun310@sjtu.edu.cn](mailto:pujun310@sjtu.edu.cn).

Feng Wang, Ph.D.; Shanghai Institute of Immunology, Department of Immunology and Microbiology, State Key Laboratory of Oncogenes and Related Genes, Shanghai Jiao Tong University School of Medicine, Shanghai, China. E-mail: wangfeng16@sjtu.edu.cn.

^#^These authors contributed equally to this work.

Supplementary Figures and Figure legends
Supplementary **Figure S1.**


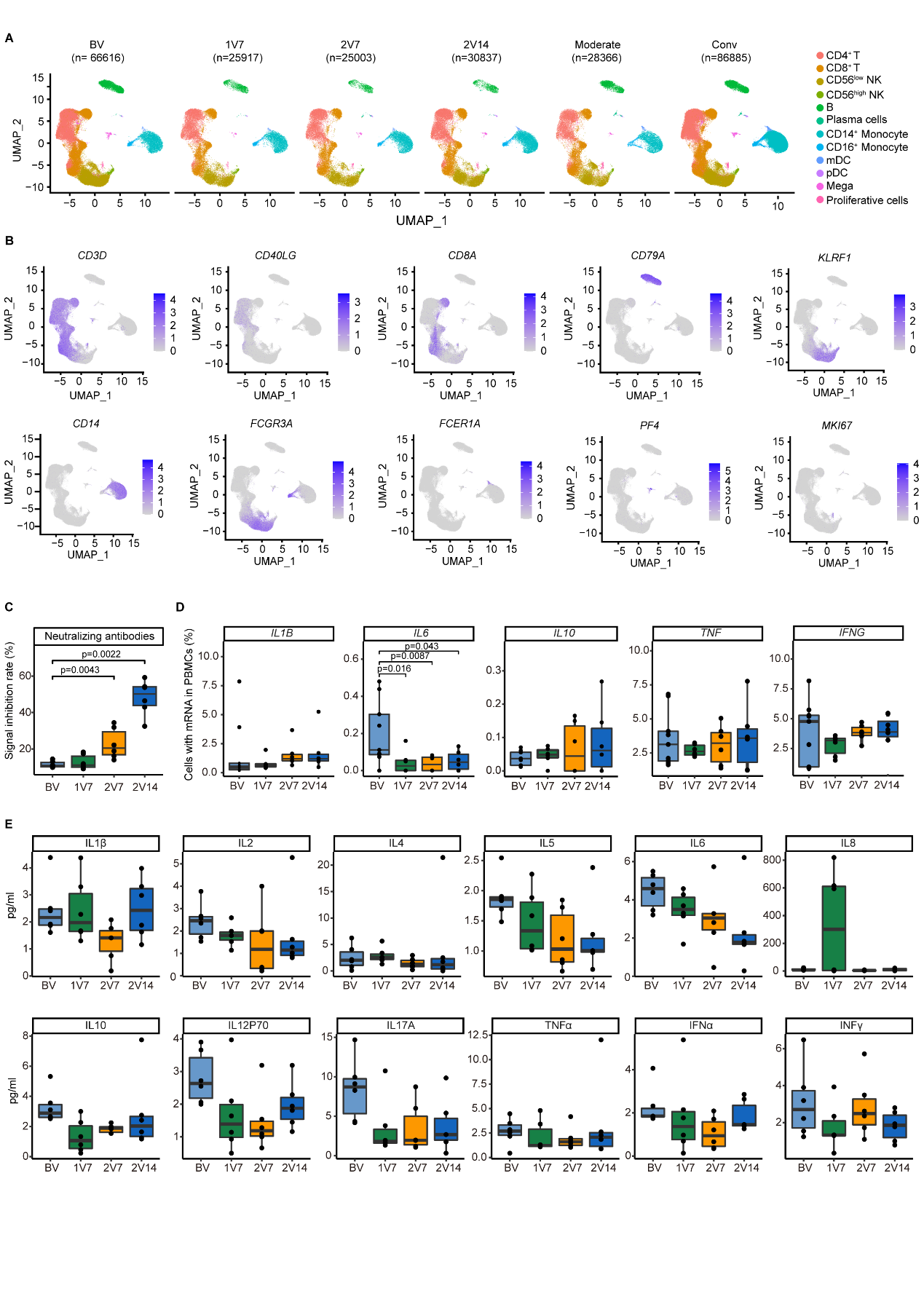


**Figure S1.** **Overview of Immunological changes with BBIBP-CorV vaccination and SARS-CoV-2 infection**

**A**. UMAP charts of 263,624 single cells grouped by six conditions. including before vaccination (BV), 7 days after the first dose (1V7), 7 days after the second dose (2V7), 14 days after the second dose (2V14), moderate patients and convalescent patients (conv). **B**. Feature plots of selected signature genes for 12 cell types. Expression levels were color-coded on the data, and the legend was labeled according to log scale. **C.** The signal inhibition rate of anti-SARS-Cov-2 RBD neutralizing antibodies across the four groups. **D**. Fraction of cells expressing *IL1B*, *IL6*, *IL10*, *TNF* and *IFNG* in BV group (n=9), 1V7 group (n=6), 2V7 group (n=6) and 2V14 group (n=6). All box plots display the median, 25th and 75th percentiles, and whiskers extending to the maximum and minimum data points. Data was analyzed by unpaired Mann-Whitney U-test. A p value< 0.05 was considered statistically significant. **E**. The key cytokines were detected at four groups using BD FortessaX20 platform (n=6 in BV, 1V7, 2V7 and 2V14 groups). All box plots display the median, 25th and 75th percentiles, and whiskers extending to the maximum and minimum data points. Conv, convalescent patients. Mega, megakaryocytes.

Supplementary **Figure S2.**


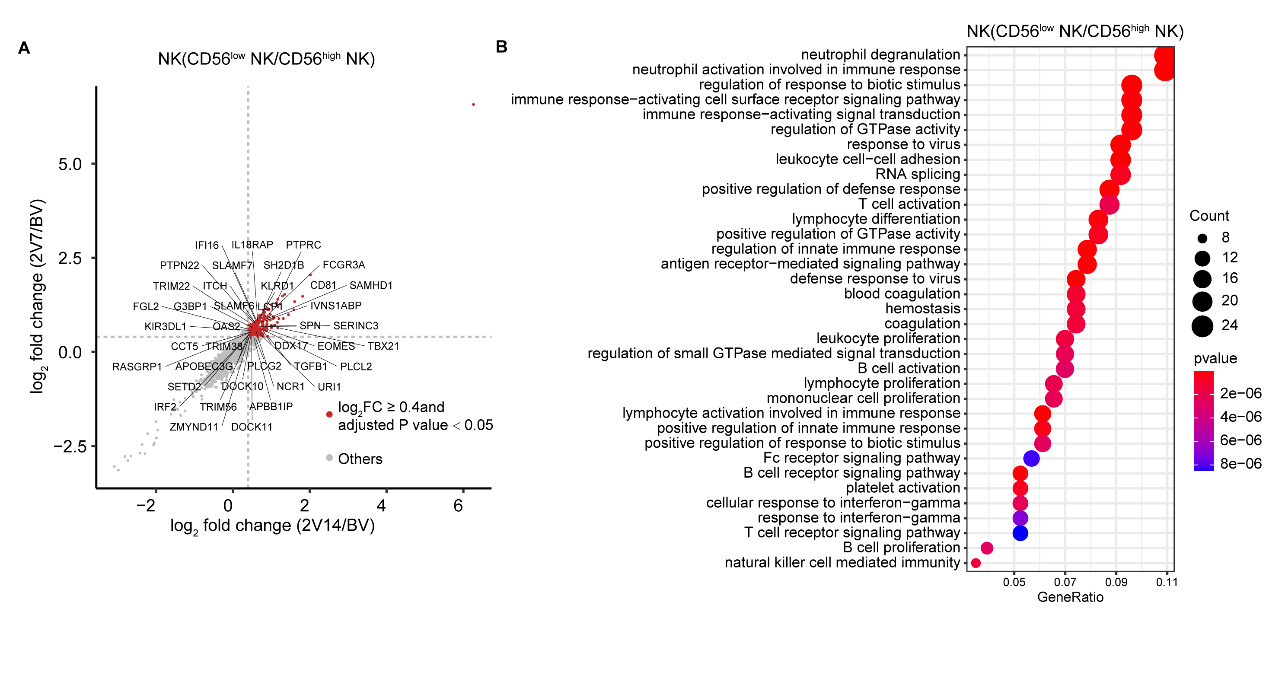
**Figure S2. Characterization of NK cells after BBIBP-CorV vaccination
A**. Scatter-plot showing DEGs in the NK cells (CD56^low^ NK and CD56^high^ NK) of the 2V7 and 2V14 groups in comparison with those of the BV group. Each red dot denoted an individual gene with Log_2_FC≥0.4 and adjusted p value<0.05 for the 2V7/BV and 2V14/BV comparisons. **B**. Functional enrichment analyses of the DEGs colored in red in scatter-plot of **A**. The top 30 enriched BP terms are shown. Interesting BP terms are highlighted in red. DEGs, differentially expressed genes. FC, fold change. BP, biological process. BV group, before vaccination. 2V7 group, 7 days after the second vaccination. 2V14 group, 14 days after the second vaccination.

Supplementary **Figure S3.**


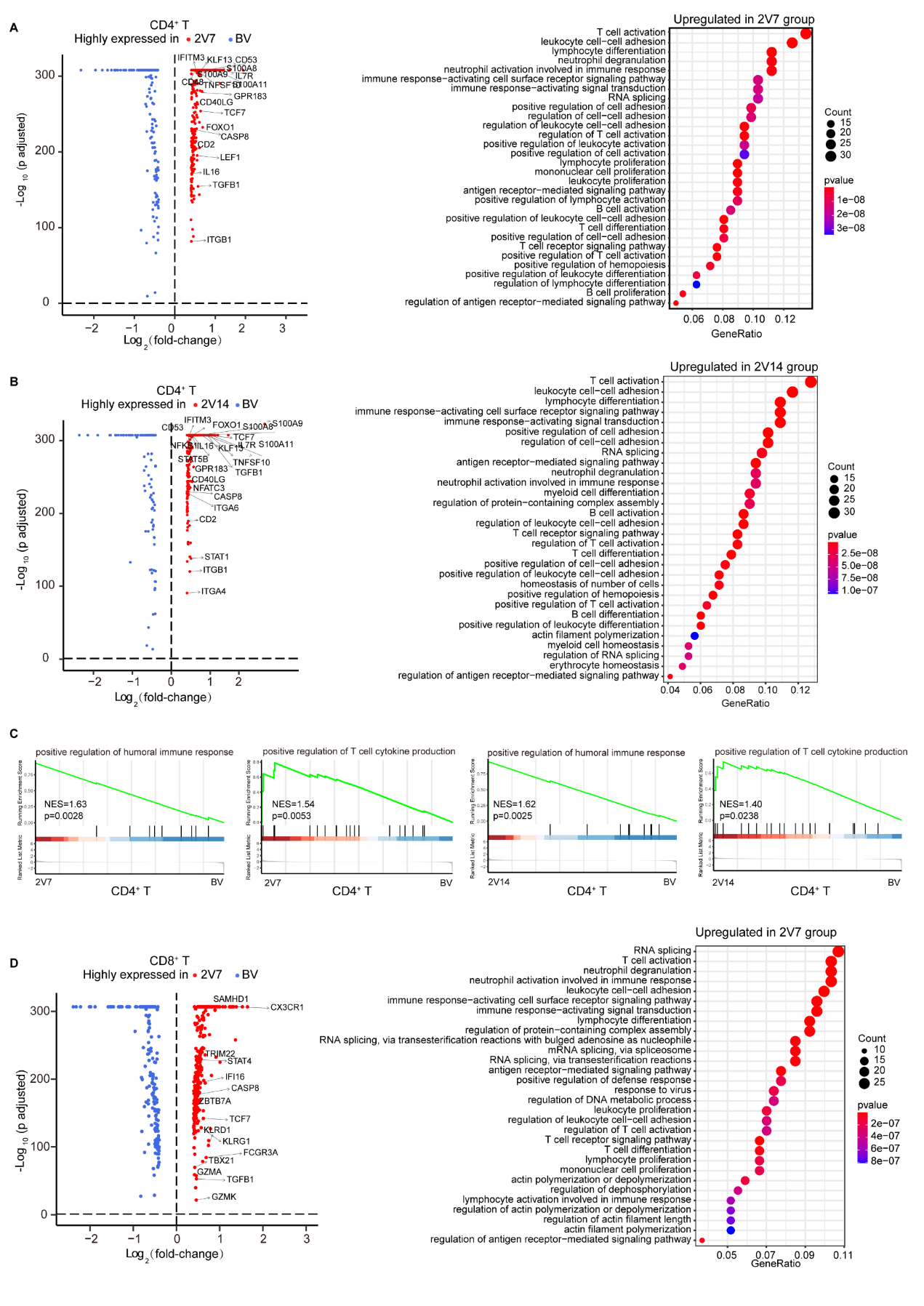


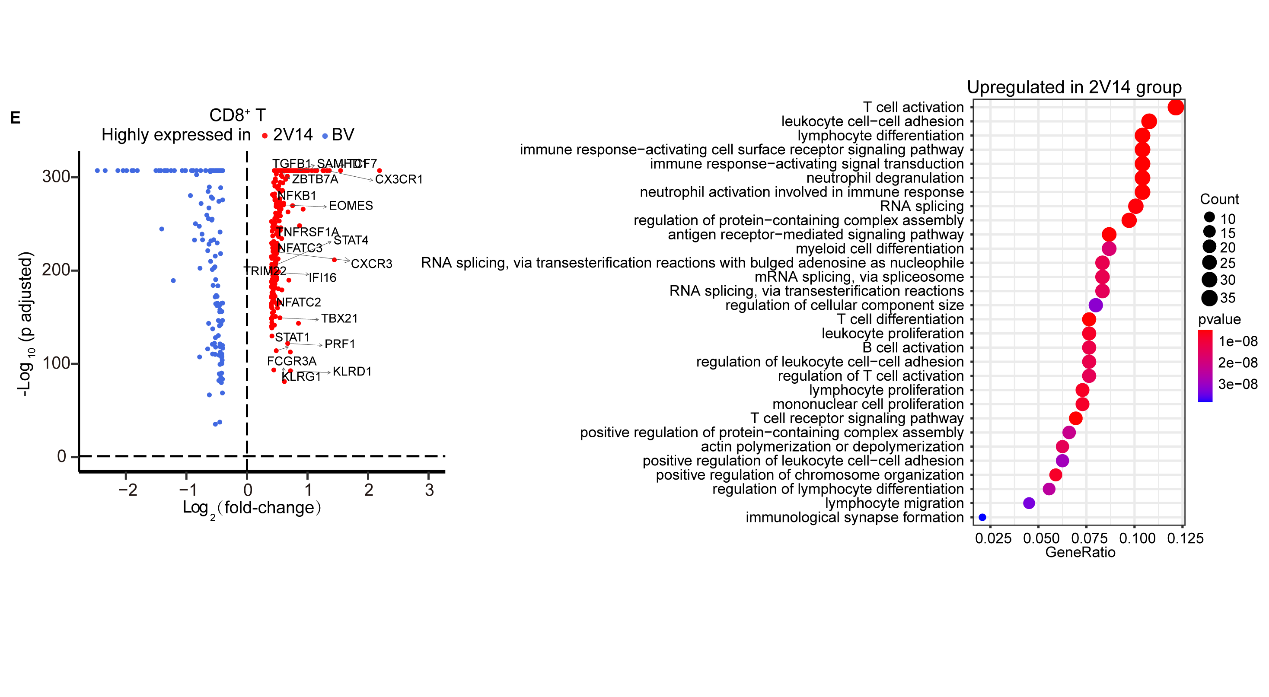


**Figure S3. Characterization of CD4^+^ T cells and CD8^+^ T cells post second dose vaccination**

**A**. Volcano plot showing the DEGs in CD4^+^ T cells of the 2V7 group in comparison with those of BV group (left panel). Example genes were labeled with gene name. Red, upregulated in the 2V7 group (Log_2_FC≥0.4, adjusted p value<0.05); blue, downregulated in the 2V7 group (Log_2_ FC≤-0.4, adjusted p value<0.05). Functional enrichment of upregulated DEGs in CD4^+^ T cells of the 2V7 group in comparison with those of BV group (right panel). The top 30 enriched BP terms are shown. **B**. Volcano plot and functional enrichment analysis similar to **A,** but for the 2V14/BV comparisons in CD4^+^ T cells. **C.** GSEA enrichment plots showing two upregulated gene sets in CD4^+^ T cells of the 2V7 and 2V14 groups in comparison with those of BV group. NES, normalized enrichment score. A p value< 0.05 was considered statistically significant. **D**. Volcano plot showing the DEGs in CD8^+^ T cells of the 2V7 group in comparison with those of BV group (left panel). Example genes are labeled with gene name. Red, upregulated in the 2V7 group (Log_2_FC≥0.4, adjusted p value<0.05); blue, downregulated in the 2V7 (Log_2_FC≤-0.4, adjusted p value<0.05). Functional enrichment of upregulated DEGs in CD8^+^ T cells of the 2V7 group in comparison with those of BV group (right panel). The top 30 enriched BP terms are shown. **E**. Volcano plot and functional enrichment analysis similar to **D,** but for the 2V14/BV comparisons in CD8^+^ T cells. DEGs, differentially expressed genes. FC, fold change. BP, biological process. BV group, before vaccination. 2V7 group, 7 days after the second vaccination. 2V14 group, 14 days after the second vaccination.

Supplementary **Figure S4.**


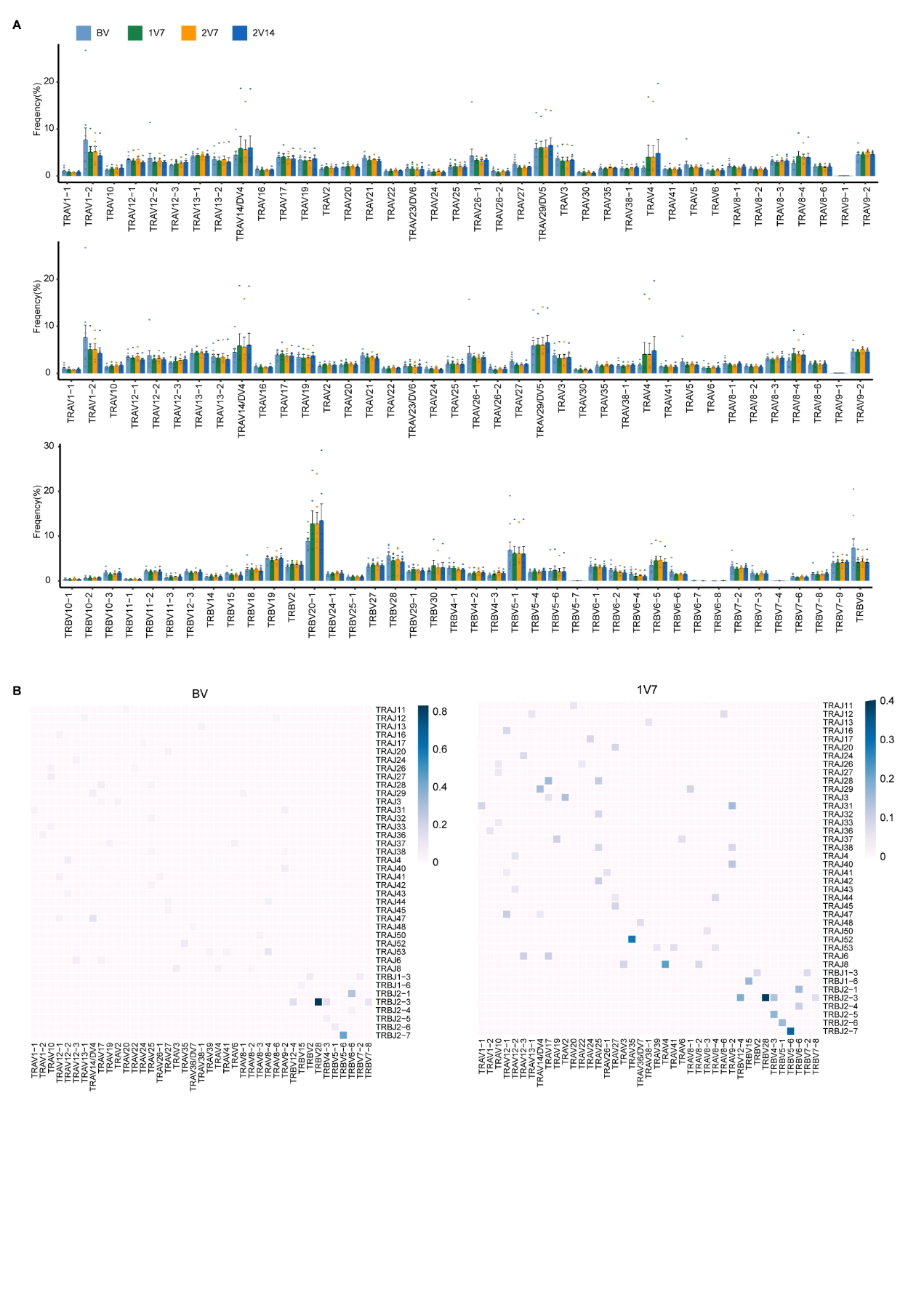


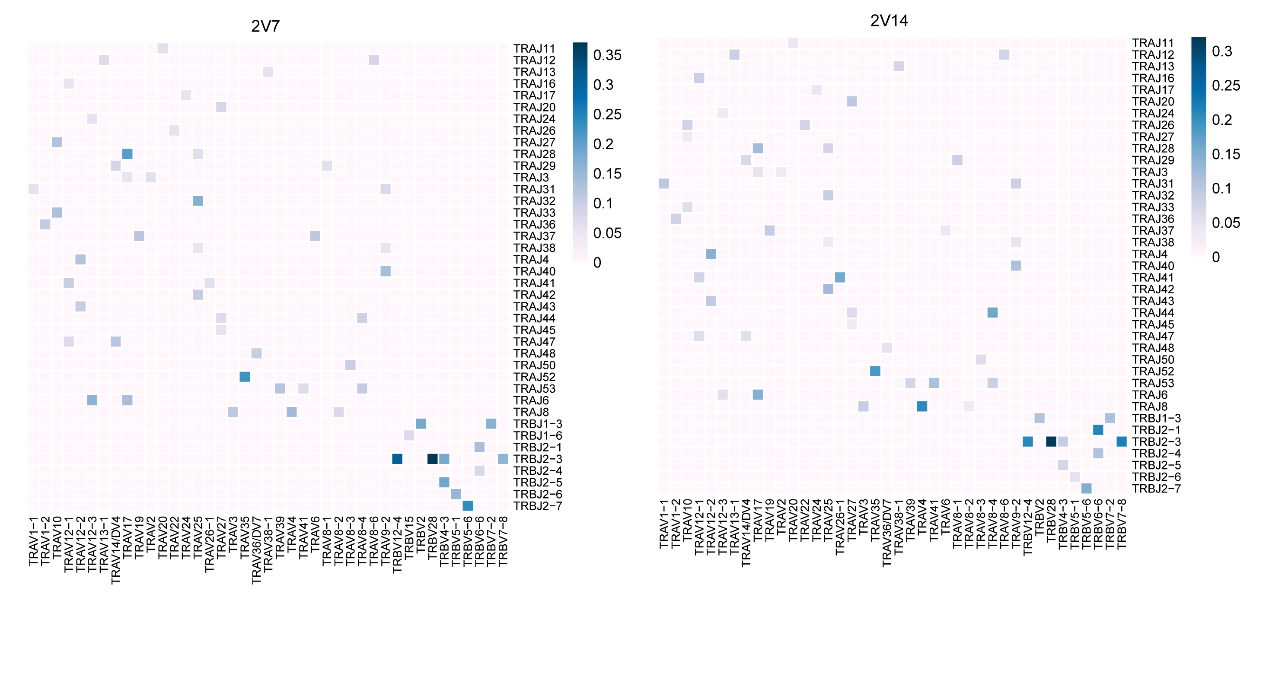


**Figure S4. VJ genes of TCR α and TCR β chains usage and rearrangement after** **BBIBP-CorV vaccination**

**A.** Bar plots showing the usage of some TRAV, TRAJ and TRBV genes across four groups (n=9 in BV group, n=6 in 1V7, 2V7 and 2V14 groups). Error bars represent ± s.e.m. **B.** Heatmap showing the TCR α and TCR β chains rearrangement in the BV, 1V7, 2V7 and 2V14 groups, respectively. The colors indicate the usage percentage of specific V-J gene pairs. BV group, before vaccination. 1V7 group, 7 days after the first vaccination. 2V7 group, 7 days after the second vaccination. 2V14 group, 14 days after the second vaccination.

Supplementary **Figure S5.**


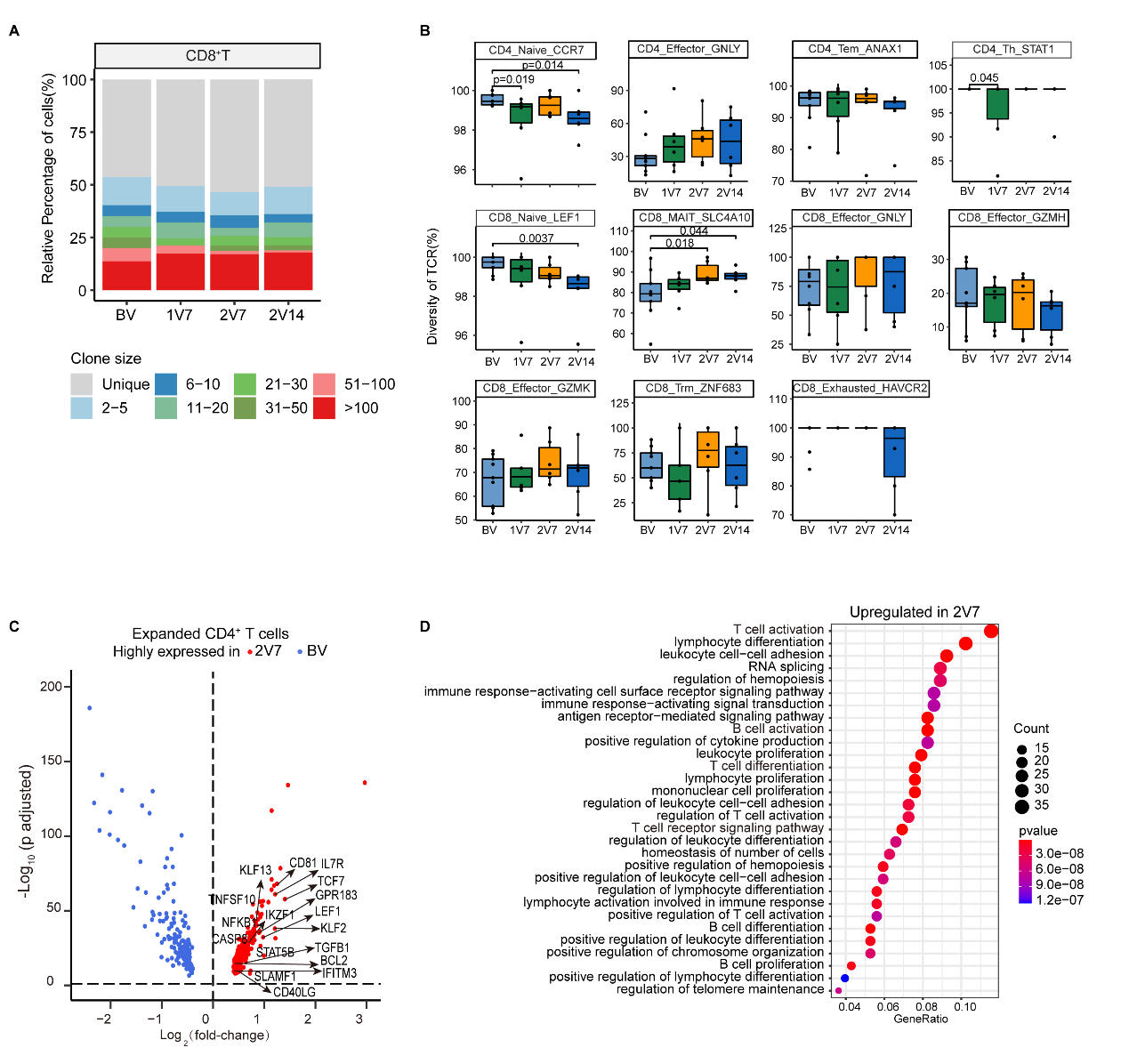


**Figure S5. T cell clonal diversity and expansion after BBIBP-CorV vaccination**

**A**. Bar plot showing the distribution of CD8^+^ T cell clone sizes across four groups. **B**.

Box plots showing the clone diversity of T cell subsets across four groups (n=9 in BV group, n=6 in 1V7, 2V7 and 2V14 groups). Groups were shown in different colors. All box plots displayed the median, 25th and 75th percentiles, and whiskers extending to the maximum and minimum data points. Data was analyzed by an unpaired Mann-Whitney U-test. A p value < 0.05 was considered statistically significant. **C**. Volcano plot showing the DEGs in the expanded CD4^+^ T cells (clone size ≥2) of the 2V7 group in comparison with those of the BV group. Example genes are labeled with gene name. Red, upregulated in the 2V7 group (Log_2_FC≥0.4, adjusted p value<0.05); blue, downregulated in the 2V7 group (Log_2_FC≤-0.4, adjusted p value<0.05). **D**. Functional enrichment analysis of upregulated DEGs in clonal expanded CD4^+^ T cells of the 2V7 group in comparison with those of BV group. The top 30 enriched BP terms are shown. DEGs, differentially expressed genes. FC, fold change. BP, biological process. BV group, before vaccination. 1V7 group, 7 days after the first vaccination. 2V7 group, 7 days after the second vaccination. 2V14 group, 14 days after the second vaccination.

Supplementary **Figure S6.**


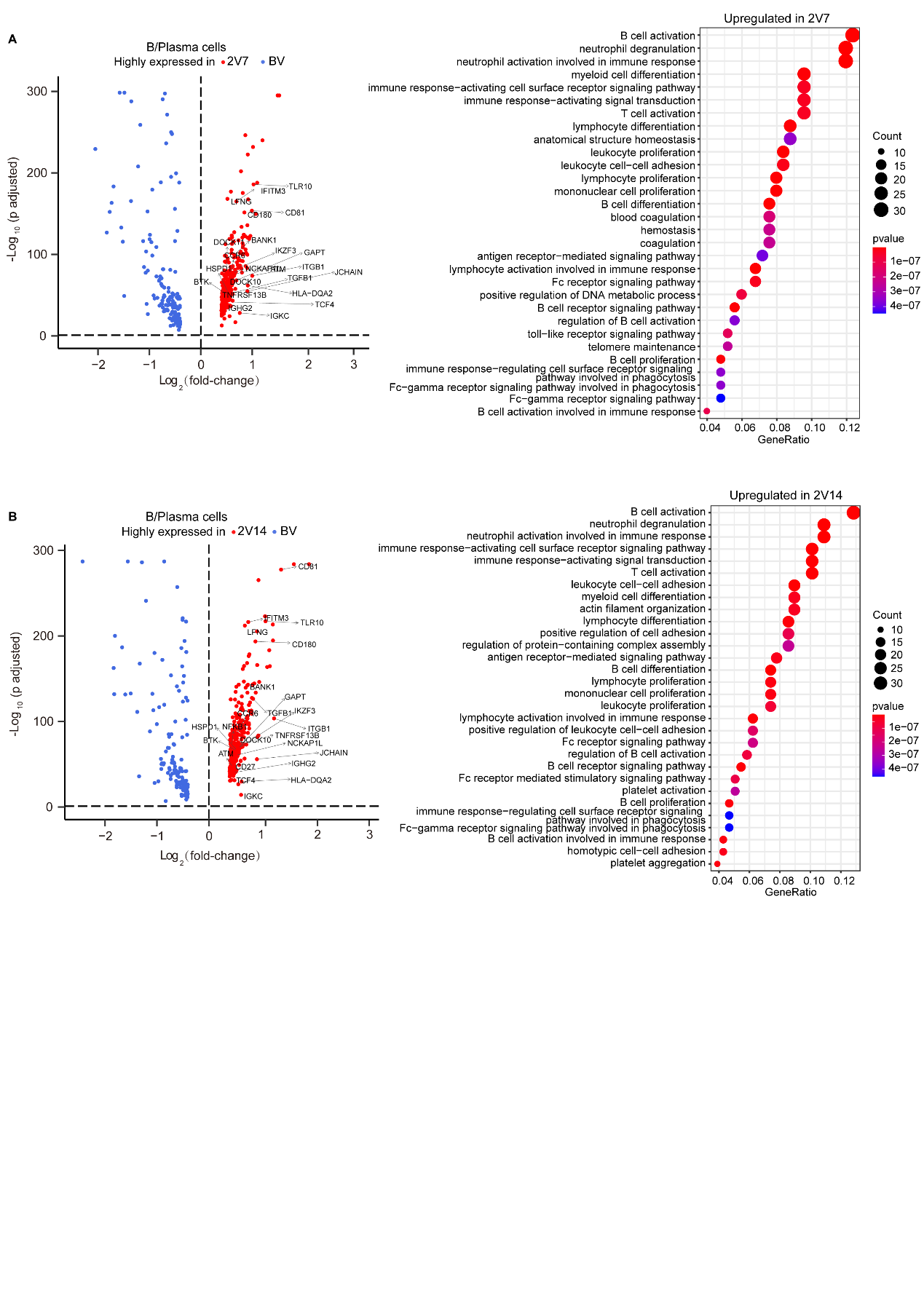


**Figure S6. DGEs analysis and functional enrichment analysis of B/plasma cells in 2V7 and 2V14 groups**

**A.** Volcano plot showing the DEGs in the B/plasma cells of 2V7 group in comparison with those of BV group (left panel). Example genes are labeled with gene name. Red, upregulated in the 2V7 group (Log_2_FC≥0.4, adjusted p value<0.05); blue, downregulated in the 2V14 group (Log_2_FC≤-0.4, adjusted p value<0.05). Functional enrichment analysis of upregulated DEGs in B/plasma cells of the 2V7 group in comparison with those of BV group (right panel). The top 30 enriched BP terms are shown. **B**. Volcano plot and functional enrichment analysis similar to **A,** but for the 2V14/BV comparisons in B/plasma cells. DEGs, differentially expressed genes. FC, fold change. BP, biological process. BV group, before vaccination. 2V7 group, 7 days after the second vaccination. 2V14 group, 14 days after the second vaccination

Supplementary **Figure S7.**


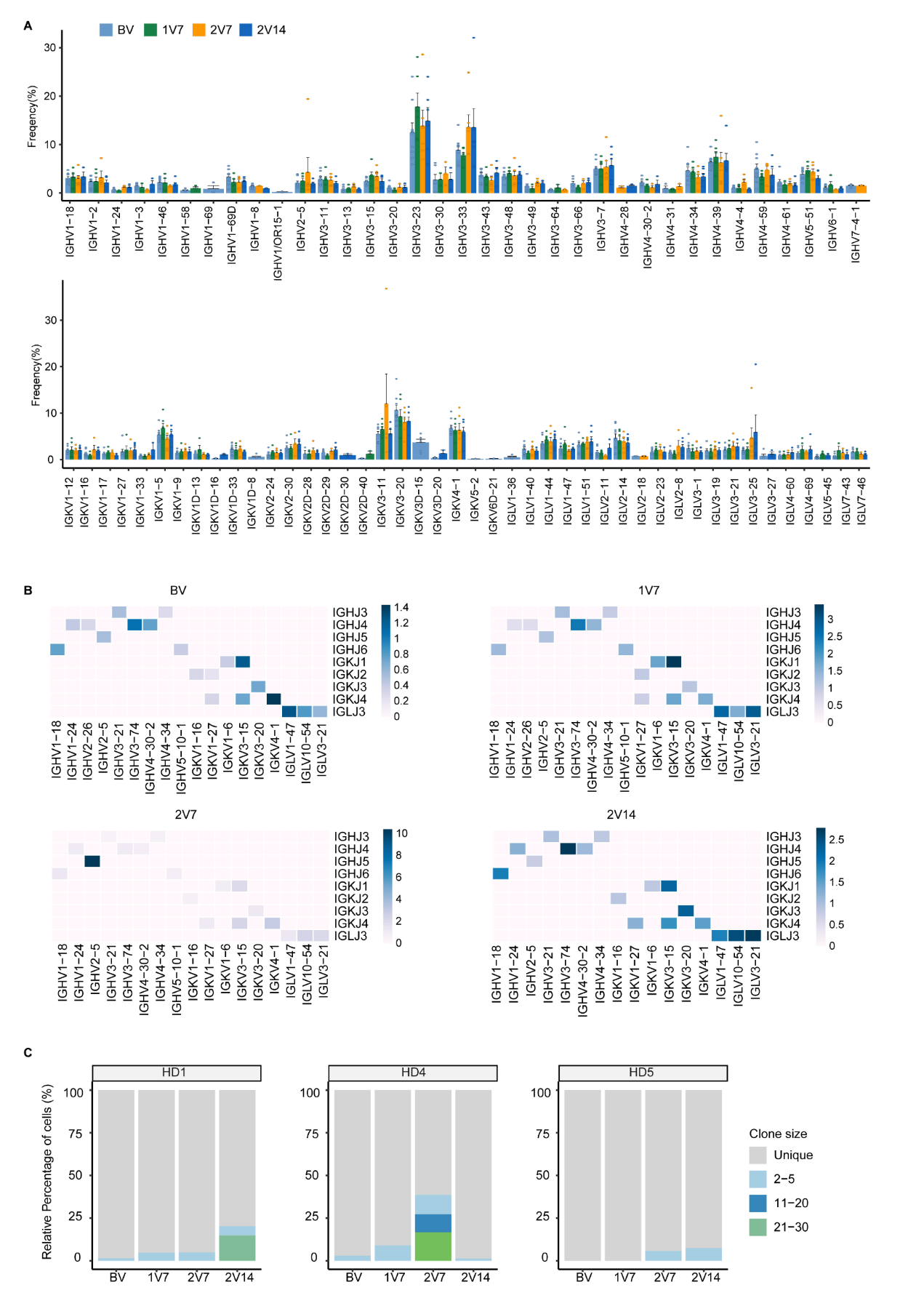


**Figure S7. VJ genes of BCR heavy and light chains usage and rearrangement after BBIBP-CorV vaccination**

**A.** Bar plots showing the usage of some IGHV, IGKV and IGLV genes across four groups (n=9 in BV group, n=6 in 1V7, 2V7 and 2V14 groups). Error bars represent ± s.e.m. **B.** Heatmap showing the IGH/K/L rearrangement in the BV, 1V7, 2V7 and 2V14 groups, respectively. The colors indicate the usage percentage of specific V-J gene pairs. **C**. Bar plot showing the distribution of B cell clone sizes across four groups derived from HD1, HD4 and HD5. HD, healthy donor. BV group, before vaccination. 1V7 group, 7 days after the first vaccination. 2V7 group, 7 days after the second vaccination. 2V14 group, 14 days after the second vaccination.

Supplementary **Figure S8.**


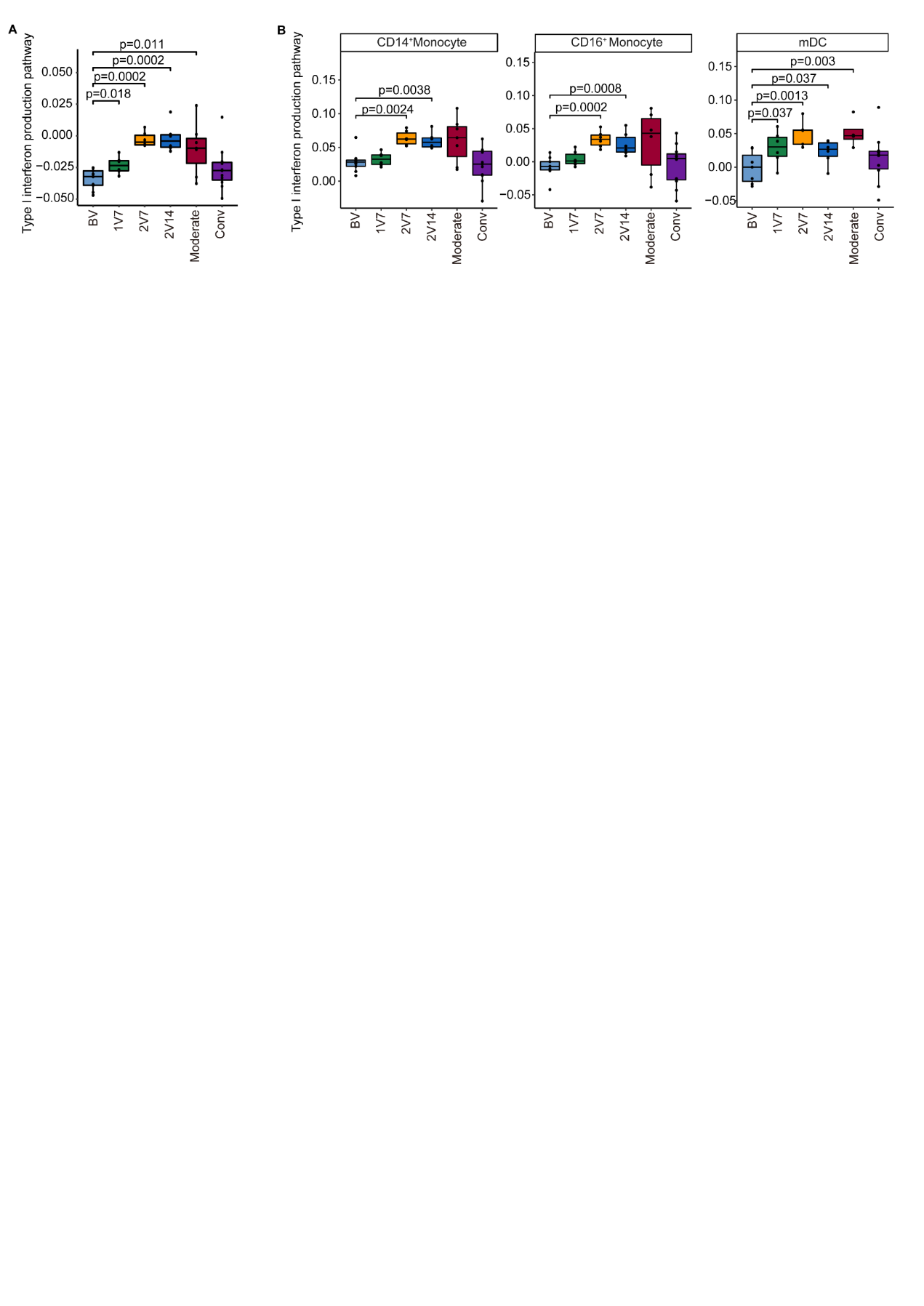


**Figure S8. Expression of type I interferon production pathway in vaccination and SARS-CoV-2 infection**

**A**. Box plots showing the expression of type I interferon production pathway across six groups (n=9 in BV, n=6 in 1V7, 2V7 and 2V14 groups, n=6 in moderate group, n=11 in conv group). Groups are shown in different colors. All box plots display the median, 25th and 75th percentiles, and whiskers extending to the maximum and minimum data points. Data were analyzed by an unpaired Mann-Whitney U-test. A p value< 0.05 was considered statistically significant. **B**. Box plots showing the expression of type I interferon production pathway in CD14^+^ Monocytes, CD16^+^ Monocytes and mDCs derived from BV group (n=9), 1V7 group (n=6), 2V7 group (n=6), 2V14 group (n=6), moderate (n=6) and conv group (n=11). Groups were shown in different colors. All box plots display the median, 25th and 75th percentiles, and whiskers extending to the maximum and minimum data points. Data was analyzed by an unpaired Mann-Whitney U-test. A p value < 0.05 was considered statistically significant. Conv, convalescent patients.

Supplementary Tables

Supplementary **Table 1. Detailed clinical characteristics of samples.**

Supplementary **Table 2. Marker genes of each cell type.**

Supplementary **Table 3. Cell number of each cell type.**

Supplementary **Table 4. Differentially expressed genes in each cell type.**

**Supplementary Table 5. Cell number of clonal expanded CD4 T cells.**
